# Targeted therapeutics and novel signaling pathways in non-alcohol-associated fatty liver/steatohepatitis (NAFL/NASH)

**DOI:** 10.1038/s41392-022-01119-3

**Published:** 2022-08-13

**Authors:** Xiaohan Xu, Kyle L. Poulsen, Lijuan Wu, Shan Liu, Tatsunori Miyata, Qiaoling Song, Qingda Wei, Chenyang Zhao, Chunhua Lin, Jinbo Yang

**Affiliations:** 1 School of Medicine and Pharmacy, Ocean University of China, Qingdao, China; 2 Department of Anesthesiology, McGovern Medical School, University of Texas Health Science Center, Houston, TX USA; 3 Innovation Center of Marine Drug Screening & Evaluation, Qingdao National Laboratory for Marine Science and Technology, Qingdao, China; 4 Department of Gastroenterological Surgery, Graduate School of Medical Sciences, Kumamoto University, Kumamoto, Japan; 5 School of Medicine, Zhengzhou University, Zhengzhou, China; 6 Department of Urology, The Affiliated Yantai Yuhuangding Hospital of Qingdao University, Yantai, China

**Keywords:** Cell biology, Endocrine system and metabolic diseases

## Abstract

Non-alcohol-associated fatty liver/steatohepatitis (NAFL/NASH) has become the leading cause of liver disease worldwide. NASH, an advanced form of NAFL, can be progressive and more susceptible to developing cirrhosis and hepatocellular carcinoma. Currently, lifestyle interventions are the most essential and effective strategies for preventing and controlling NAFL without the development of fibrosis. While there are still limited appropriate drugs specifically to treat NAFL/NASH, growing progress is being seen in elucidating the pathogenesis and identifying therapeutic targets. In this review, we discussed recent developments in etiology and prospective therapeutic targets, as well as pharmacological candidates in pre/clinical trials and patents, with a focus on diabetes, hepatic lipid metabolism, inflammation, and fibrosis. Importantly, growing evidence elucidates that the disruption of the gut–liver axis and microbe-derived metabolites drive the pathogenesis of NAFL/NASH. Extracellular vesicles (EVs) act as a signaling mediator, resulting in lipid accumulation, macrophage and hepatic stellate cell activation, further promoting inflammation and liver fibrosis progression during the development of NAFL/NASH. Targeting gut microbiota or EVs may serve as new strategies for the treatment of NAFL/NASH. Finally, other mechanisms, such as cell therapy and genetic approaches, also have enormous therapeutic potential. Incorporating drugs with different mechanisms and personalized medicine may improve the efficacy to better benefit patients with NAFL/NASH.

## Introduction

Hepatic steatosis (fatty liver) is one of the most prevalent chronic liver diseases worldwide, affecting approximately one quarter of the global population, and is predicted to become the leading indication for liver transplantation by 2030, posing a significant burden on global health.^1–6^ According to the history of alcohol intake, fatty liver is artificially categorized into two common forms: alcohol-associated liver disease (ALD) and non-alcohol-associated fatty liver/steatohepatitis (NAFL/NASH).^4,7–10^ While ALD is defined by the presence of hepatic steatosis associated with significant alcohol consumption, NAFL is a generic term that includes a series of liver diseases with different injury severities and consequent fibrosis.^4,9^ Among these, hepatic steatosis is referred to as NAFL, which is defined as the composition of fat that takes up 5–10% of the liver’s weight. NASH is associated with inflammation and fibrosis, which may progress to cirrhosis and hepatocellular carcinoma (HCC).^11–14^ About 20% of patients with NAFL develop NASH, and over 40% of patients with NASH progress to fibrosis.^15,16^ However, HCC can also develop in the absence of cirrhosis.^17,18^

Fatty degeneration of the liver, as a pathological change, was first proposed by William Bowman who found that fat accumulation in the liver through observing human liver specimens under the microscope in 1842.^19^ For the next hundred years, it was generally believed that long-term alcohol consumption was the major cause of the fatty liver; however, a considerable proportion of fatty liver was identified in obese and diabetic people without drinking history.^20,21^ In 1980, Dr Jurgen Ludwig first proposed the concept of NASH^22^ and Dr Fenton Schaffner suggested the concept of non-alcohol-associated fatty liver disease (NAFLD) in 1986.^23^ The term NAFLD has evolved throughout history with advances in the understanding of disease pathophysiology and diagnostic methods.^14^ NASH is the subtype of NAFLD that can culminate in cirrhosis, HCC, and even death.^24^ However, the molecular mechanisms underlying the transition from NAFL to NASH are complex and not yet fully understood.^13^ NAFL/NASH, as a multisystem metabolic disease, is also associated with extrahepatic organ diseases, such as cardiovascular disease (CVD),^25,26^ chronic kidney disease (CKD),^27,28^ dementia, and sleep apnea.^29,30^ Despite increasing liver-related mortality, CVD remains the primary cause of death in patients with NAFL/NASH.^16^

### Clinical progression of NAFL/NASH

Although NAFL/NASH develops at different rates among individuals, it typically follows four stages.^31^ The first stage involves liver fat accumulation, also known as NAFL. The second stage is referred to as early NASH (F0 no fibrosis and F1 negligible fibrosis) and is characterized by fatty infiltration and liver inflammation. The diagnosis of NASH requires the presence of steatosis, ballooning, and lobular inflammation in liver biopsy. Other histological changes, including portal inflammation, polymorphonuclear infiltrates, Mallory–Denk bodies, apoptotic bodies, clear vacuolated nuclei, microvacuolar steatosis, and megamitochondria, can be seen in NASH, but are not necessary for the diagnosis.^7^ The third stage, known as fibrosis (F2 advanced fibrosis and F3 bridging fibrosis), is caused by chronic liver inflammation and injury, which results in the excessive accumulation of extracellular matrix (ECM) proteins, including collagen, in the liver. The fourth stage is liver cirrhosis (F4), a severe stage of NAFL/NASH that can be life threatening and develop into end-stage liver disease (ESLD), which is fatal without a transplant.^4,7,15,32^

### NAFL/NASH-related epidemiology

The prevalence of NAFL/NASH is parallel with age, the development of obesity, and type 2 diabetes mellitus (T2DM), and it varies with country and ethnicity.^15^ Globally, it is estimated that NAFL/NASH accounts for approximately 25% of the general population. By 2030, this percentage is expected to increase, and the proportion of patients with NAFL/NASH affected by terminal diseases will be even higher.^3,6,15^ Notably, in T2DM patients, the global prevalence of NASL/NASH is two-fold higher than in the general population, amounting to 55.5%, and the highest prevalence was reported in Europe (68%).^33^ The global prevalence of NASH among patients with T2DM is 37.3%. Approximately 17% of patients with NAFL/NASH and T2DM have developed advanced fibrosis.^33^ Age affects the incidence of NAFL/NASH, with the mean age of 70–79 having the highest prevalence (33.99%), followed by 60–69 (28.9%), 50–59 (27.4%), and 40–49 (26.53), and 30–39 holding the lowest prevalence (22.43%).^15^ A study has revealed that lipid turnover, the balance between lipid storage and removal, in adipose tissue decreases with age, whether weight loss or gain.^34^ The decrease in lipid turnover rate was associated with insulin resistance (IR), dyslipidemia, and metabolic disorders that could increase the risk of obesity, NAFL/NASH, and other chronic diseases.^35^ Furthermore, the frequency and severity of NAFL/NASH vary by geographic region and ethnicity. Specifically, the Middle East was found to have the greatest frequency of NAFL/NASH (31.79%), followed by Asia (27.37%), South America (24.13%), North America (24.13%), and Europe (23.71%), while Africa had the lowest prevalence (13.48%).^4,15^ The prevalence in different regions is closely related to their genetic background, lifestyle, and economic status. Current estimates of direct medical costs for NAFLD exceed $100 billion annually in the United States, with the majority of that spent on NASH and its subsequent diseases.^36^ In addition to cirrhosis and HCC, NAFL/NASH significantly increases the incidence of multiple extrahepatic complications such as T2DM, CVD, CKD, and some extrahepatic malignancies.^37^ Patients with NAFL/NASH had a 64% increased risk of CVD, and the incidence of CVD is proportional to the severity of NAFL/NASH.^38^ Patients with NAFL/NASH also develop coronary atherosclerosis, myocardial alterations, and arrhythmias, all of which raise the risk of heart failure.^39^ NAFL/NASH also significantly increases the risk of extrahepatic cancers such as colorectal tumors,^40^ gastric cancer,^41^ pancreatic cancer,^42^ uterus cancer,^43^ and breast cancer.^44^ Hence, it is important to design effective treatment for metabolic syndrome and cancer screening programs for patients with NAFL/NASH. It is also needed to take effective interventions to prevent and control the prevalence of NAFL/NASH to reduce the economic and social burden.

**Fig. 1:**
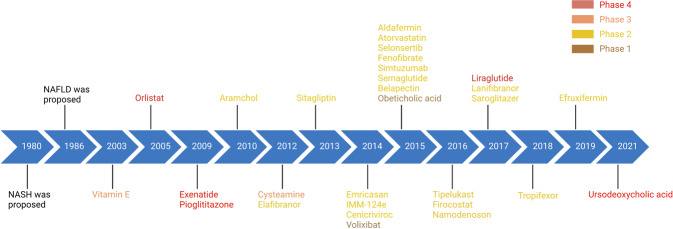
Timeline of NAFL/NASH-related drug development. Drugs at different clinical stages are indicated in different colors: phase 4 drugs are marked in red, phase 3 drugs are marked in orange, phase 2 drugs are marked in yellow, phase 1 drugs are marked in brown, and preclinical drugs are marked in cyan. All colors of drugs in the following figures are the same. Created with BioRender

Currently, there are no approved treatments specific to NAFL/NASH despite the high incidence and growing global health impact. While steady progress has been made in the understanding of NAFL/NASH pathophysiology and identification of therapeutic targets, relatively slow progress was achieved in the treatment of all aspects of NAFL/NASH even after years of intense research,^5,13,45^ although several approved drugs for treating metabolic-related disorders and diseases showed promising outcomes in patients with NAFL/NASH, including Orlistat^46^ used for the treatment of obesity (Fig. 1). Appropriate therapeutic targets and potent drug candidates are urgently demanded. Herein, we highlight the current understanding of the pathogenesis of NAFL/NASH and outline potential therapeutic targets and corresponding drug candidates in preclinical/clinical trials or patents for treating NAFL/NASH. These emerging therapies namely target diabetes, hepatic lipid metabolism, inflammation, and fibrosis. In addition, advanced research on the signaling pathways that participate in NAFL/NASH pathogenesis has been recognized, including extracellular vesicles (EVs) and gut microbiota, which may provide more rationales and strategies for individualized approaches for future management of NAFL/NASH.

## Signaling pathways driving NAFL/NASH development and related therapeutic targets

**Fig. 2:**
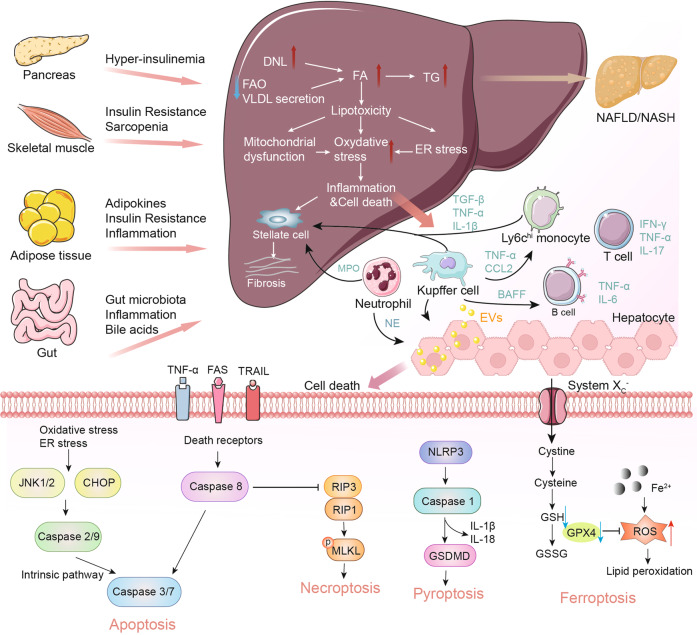
Schematic summary of the pathogenesis and interorgan crosstalk of NAFL/NASH. Increased lipid synthesis and uptake in the liver exceeds lipid oxidation and excretion, leading to lipid accumulation and lipotoxicity, inflammatory response, cell death, and fibrosis. Besides the liver, insulin-sensitive organs, such as adipose tissue and muscle, produce adipokines and myokines, respectively, which promote inflammation and oxidative stress in the liver. The gut microbiota regulates the inflammatory response and hepatic lipid accumulation through the metabolism of PAMPs, bile acids, etc. Innate immune responses involved in NAFL/NASH include activation of resident Küpffer cells and recruitment of leukocytes (e.g., neutrophils, monocytes) to the liver. Lymphocyte-mediated adaptive immunity is an additional factor promoting liver inflammation. EVs act as drivers of inflammation in NAFL/NASH activating immune cells and HSC. In NAFL/NASH progression, lipotoxicity-induced hepatocyte death is an important driver including apoptosis, necroptosis, pyroptosis, and ferroptosis. Arrows (red) indicate upregulation and arrows (blue) indicate downregulation in NAFL/NASH. Produced with the assistance of Servier Medical Art (https://smart.servier.com). DNL de novo lipogenesis, FA fatty acid, FAO fatty acid oxidation, TG triglyceride, VLDL very-low-density lipoprotein, ER endoplasmic reticulum, MPO myeloperoxidase, NE neutrophil elastase, BAFF B cell-activating factor, TGF-β transforming growth factor beta, TNF-α tumor necrosis factor-alpha, IL interleukin, IFN interferon, CCL2 C-C motif ligand 2, TRAIL tumor necrosis factor-related apoptosis-inducing ligand, CHOP C/EBP homologous protein, RIP receptor-interacting serine-threonine kinase, MLKL mixed lineage kinase domain-like protein, NLPR3 NACHT, LRR, and PYD domains-containing protein 3, GSDMD gasdermin D, GSH glutathione, GSSG glutathione disulfide, GPX4 glutathione peroxidase 4, ROS reactive oxygen species, NAFL nonalcoholic fatty liver, EVs extracellular vesicles

The development of NAFL/NASH is considered to initiate from simple steatosis as the first hit that is not enough to induce inflammation and fibrosis; however, during disease progression, a following second hit, including oxidative stress, is necessary to aggravate liver damage.^47^ NASH is the result of multiple factors acting simultaneously, including genetic variants, abnormal lipid metabolism, oxidative stress, altered immune response, and imbalances in the gut microbiota.^48^ The “multiple hits” implies that liver inflammation, instead of steatosis, is the primary cause of NASH progression to fibrosis, therefore probably multiple mechanisms act in synergy to promote disease progression.^49^ The substrate-overload lipotoxic liver injury model of NAFL/NASH revealed that the liver is overwhelmed in dealing with the primary metabolic energy substrates, carbohydrates, and fatty acids, which subsequently lead to the accumulation of toxic lipid species.^50–52^ These metabolites can further induce hepatocellular stress, injury, and hepatic death, resulting in fibrogenesis and genomic instability that make patients susceptible to cirrhosis and HCC (Fig. 2).^5,53^ While numbers of the current drugs in clinical trials generally achieved the effect of improving NASH histopathology (hepatic steatosis, etc.) or without worsening fibrosis, future studies are needed to translate into appropriate clinical applications.^54^ On the other hand, patients may exhibit different NAFL/NASH phenotypes due to unique genetic predispositions and idiosyncrasies within the disease, a single treatment is unlikely to reverse NAFL/NASH across all patients, pharmacological combinations and personalized therapy will be favored in the future.^53,55–57^ Here we focus on the signaling pathways that drive NAFL/NASH pathogenesis and summarize the relevant agents and therapies.

### Lifestyle interventions

Lifestyle interventions, including dietary change, exercise, and weight loss, are the major treatment strategies for NAFL patients without fibrosis development.^58^ So far, weight loss is the key to improve the histopathological features of NASH, with a clear dose-response association. It was reported that weight loss of at least 3–5% could improve hepatic steatosis, and 5–7% weight loss was necessary to reduce inflammatory activity. In addition, a weight loss of more than 10% indicated the regression of fibrosis.^59^

A prospective study evaluated the impact on patients with histologically proven NASH of lifestyle changes through a hypocaloric diet (750 kcal/d, calorie deficit) combined with exercise (walk 200 min per week) to reduce weight over 52 weeks. Paired liver biopsies showed the greater weight loss caused by lifestyle changes was related to the improvement of NASH histological characteristics. Among the patients with weight loss ≥10%, the rates of NAFLD activity score (NAS) reduction, NASH, and fibrosis regression were the highest.^60^ In addition, a small randomized controlled trial using a combination of diet, exercise, and behavior modification for 48 weeks showed that histology of the lifestyle intervention group improved significantly at 48 weeks, of which 67% have improved NAS. Liver steatosis, lobular inflammation, and ballooning in the intervention group were also improved. However, no improvement was observed in fibrosis.^61^ In addition, the clinical practice guidelines published by the European Association for the Study of the Liver, European Association for the Study of Diabetes, and European Association for the Study of Obesity recommended that the Mediterranean diet (MD) pattern, which contains high amounts of whole grains and monounsaturated fatty acids (MUFAs), as the first-choice diet for patients with NAFL/NASH.^62^ MD showed improved hepatic steatosis and reduced visceral fat in patients with NAFL in both adults and adolescents,^63,64^ and persistent MD might reduce the prevalence of NAFL and improve IR in patients with NAFL.^65^ MD was also shown to reduce platelet activation and hepatic collagen deposition, reducing the risk of CVD in patients with NAFLD.^66^ The fiber and polyphenols in whole grains of MD reduced energy intake and increased *Lactobacillus* and *Bifidobacterium* in the gut that is beneficial for improving NAFLD.^67,68^ Some butyrate-producing bacteria also increased that is beneficial for improving NAFLD.^68^ Recent research showed that a green-MD, which contains more green plants and polyphenols and less red or processed meat, led to double intrahepatic fat loss in patients with NAFLD compared with traditional MD.^69^ However, it is important to note that existing studies have focused on early NAFLD or NAFLD prevention; whether MD is effective for individuals with NASH and advanced disease states requires further investigation.

Clinical trials showed the remission of steatosis occurs with weight reduction achieved by lifestyle interventions, which remains the cornerstone of treatment. However, the effectiveness of lifestyle modification is still limited by difficulties in implementing lifestyle changes, as patients with NAFL/NASH may lack preparation for changing and adopting a healthier lifestyle, particularly regarding physical activity.^70^ Moreover, the life quality of the patients could be persistently affected by advanced symptoms and diseases, such as hepatic fibrosis, cirrhosis, and HCC, special focus should be made on pharmacological treatment in addition to lifestyle-related interventions.^58^

### Pharmacological interventions

Although lifestyle interventions have been shown to improve fatty liver in NAFL patients, advanced disease conditions, such as substantial fibrosis, are unlikely to be cured by simple lifestyle interventions; therefore, pharmacological interventions remain highly demanded. Here we summarize the progress of pharmacological intervention strategies and their respective pursuant signaling pathways, including targeting metabolism, cellular stress, inflammation, and fibrosis.

#### Glucose and lipid metabolisms

**Fig. 3:**
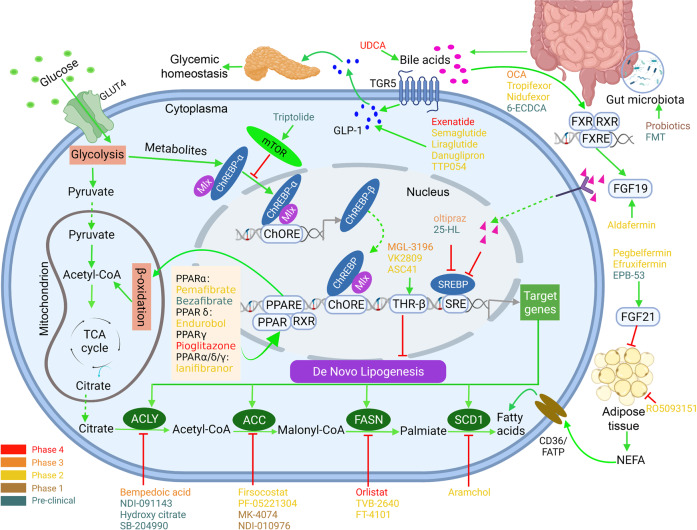
Glucose and lipid metabolisms and targeting drugs for NASH. Depiction of the drugs actions sites that are currently in preclinical and clinical trials, based on their primary locus of activity. Targets include those that regulate lipids and glucose homeostasis, such as GLP-1 signaling, mTOR signaling, PPAR signaling, BAs metabolism, DNL and NEFA metabolism, and gut microbiota targets in humans. Agonists are indicated with a green arrow and antagonists with a red inhibitor. Drugs at different clinical stages are as indicated. Created with BioRender. ACLY ATP-citrate lyase, ACC acetyl-coenzyme A carboxylase, FASN fatty acid synthase, SCD stearoyl-CoA desaturase, GLP glucagon-like peptide, FGF fibroblast growth factor, NEFA non-esterified fatty acid, FXR farnesoid X receptor, RXR retinoid X receptor, THR thyroid hormone receptor, mTOR mammalian target of rapamycin, PPARα/δ/γ peroxisome proliferator-activated receptors PPARα, PPARδ, and PPARγ, BAs bile acids, ChREBP carbohydrate response element-binding protein, SREBP sterol regulatory element-binding protein, TCA tricarboxylic acid, FMT fecal microbiota transplantation, OCA obeticholic acid, UDCA ursodeoxycholic acid

Metabolic disorders, such as steatosis, are considered essential steps in the pathogenesis of NAFL/NASH (Fig. 3), targeting the abnormal fatty acid and glucose metabolism to prevent liver fat accumulation and the production of a profibrotic environment appear to be promising therapeutic strategies.^71,72^ Here we review some of the most promising therapeutic targets for NASH, and describe compounds being evaluated against these targets in clinical or preclinical stages (Tables 1 and 2).

**Table 1:** Antidiabetic and lipid metabolism drugs under clinical trials

Class	Drug classification	Drug name	Registered clinical trails	Outcome	Ref.
Antidiabetic drugs (PPAR signaling)	PPARγ agonists	Pioglitazone	Phase 4 (NCT00994682)	↓Liver fibrosis ↓Insulin resistance ↓Liver TG	^140,141^
	PPARα/δ agonists	Elafibranor (GFT505)	Phase 3 (NCT02704403) Phase 2b (NCT01694849)	↓Liver fibrosis ↓Inflammation ↓Liver enzymes, lipids, glucose profiles	^143^
	PPARα/γ agonists	Saroglitazar	Phase 2 (NCT03061721)	↓Insulin resistance ↓Liver fibrosis ↓Plasma ALT, liver fat content	^573^
	PPARα agonists	Pemafibrate (K-877)	Phase 2 (NCT03350165)	↓MRE-based liver stiffness. ↓ ALT, LDL-C No decrease in liver fat content	^574^
		Fenofibrate	Phase 2 (NCT02781584, NCT02354976)	↓Serum triglycerides No decrease in liver fat	^575,576^
	PPARδ agonists	Endurobol (GW501516)	Phase 2 (NCT00388180)	↓VLDL, LDL, IDL ↑HDL	^577,578^
		Seladelpar	Phase 2 (NCT03551522)	No effect on hepatic steatosis ↓ALT, AST, GGT, AP	^579^
	PPARα/δ/γ agonists	Lanifibranor (IVA337)	Phase 3 (NCT04849728, recruiting) Phase 2b (NCT03008070)	↓Liver fibrosis ↓Hepatic steatosis ↓Liver enzyme levels ↓Inflammation	^146^
Antidiabetic drugs (GLP-1 agonists)	GLP agonist	Exenatide	Phase 4 (NCT01208649, NCT02303730) Phase 2 (NCT00529204)	↓Hepatic steatosis ↓ALT, AST, GGT ↓Blood glucose	^580,581^
		Liraglutide	Phase 2 (NCT01237119, NCT02654665)	↓Hepatic steatosis ↑Glucose control ↓Body weight	^154,156,582^
		Semaglutide	Phase 2 (NCT02453711, NCT02970942)	↓Hepatic steatosis ↓Body weight	^158,162^
	Glucagon/GLP-1 dual agonist	BI 456906	Phase 2 (NCT04771273, recruiting)	No results posted	
	GIP/GLP-1 receptor agonist	Tirzepatide (LY3298176)	Phase 2 (NCT03131687)	↓ALT, AST ↓Keratin-18, procollagen III ↑Adiponectin	^163,164^
	GLP-1-glucagon-GIP receptor tri-agonist	HM15211	Phase 1 (NCT03744182) Phase 2 (NCT04505436, recruiting)	↓Body weight	^583^
	Balanced glucagon-GLP-1 receptor agonist	Cotadutide	Phase 2b (NCT03235050) Phase 2 (NCT04019561, ongoing)	↓Liver fibrosis ↓Hepatic steatosis ↓Body weight ↓ALT, AST	^166^
	GCGR and GLP-1R dual agonist	Oxyntomodulin (OXM, JNJ-6456511)	Phase 1 (NCT01055340 NCT01373450)	↓Body weight ↓Serum TC, TG, LDL-C ↑Insulin secretion	^584–586^
	DPP-4 inhibitor	Sitagliptin	Phase NA (NCT01260246)	↓Hepatic steatosis ↓Body weight ↓ALT, AST, GGT, LDL	^587^
	SGLT2 inhibitors	Empagliflozin	Phase 4 (IRCT20190122042450N3, NCT02637973, NCT02686476, NCT02964715) Combined therapy (NCT04639414, NCT03646292)	↓Liver fibrosis ↓Hepatic steatosis ↓Insulin resistance ↓ALT, AST	^178–180,588^
		Dapagliflozin	Phase 3 (NCT03723252, recruiting) Phase 2 (NCT02279407) Phase 1 (NCT02696941)	↑Glucose control ↓Body weight, abdominal fat ↓ALT, AST, GGT ↓CK18-M30, CK18-M65, FGF21	^184,589^
		Dapagliflozin	UMIN000022155 UMIN000023574	↓Liver fibrosis ↓Hepatic steatosis ↓Body weight ↓ALT, GGT	^182,183^
		Canagliflozin	UMIN000023044 UMIN000020615	↓Hepatic steatosis ↓Insulin resistance ↓Body weight ↓ALT, AST, GGT	^181,590^
		Ipragliflozin	UMIN000015727 UMIN 000022651 jRCTs071180069 UMIN000029697	↓Liver fibrosis ↓Body weight, abdominal fat ↓ALT, AST, HbA1c	^176,177^
		Tofogliflozin	jRCTs031180159	↓Hepatic steatosis ↓Body weight ↓ALT, AST, GGT, HbA1c ↓CK18-M30, ↑HDL	^591^
		Luseogliflozin	UMIN000016090	↓Hepatic steatosis ↓Body weight	^592^
Lipid metabolism	Acetyl-ACC inhibitor	Firsocostat (GS-0976)	Phase 2 (NCT02856555) Phase 2b (NCT03449446)	↓Liver fibrosis ↓Hepatic steatosis ↓ALT	^85,86,570^
		PF-05221304	Phase 2a (NCT03248882) combined therapy (NCT03776175, NCT04321031, recruiting)	↓Hepatic steatosis ↑Plasma TG (co-administration with a DGAT2 inhibitor could address increased TG)	^88^
		MK-4074	Phase 1 (NCT01431521)	↓Hepatic steatosis ↑Plasma TG	^87^
	Allosteric inhibitor of ACC1 and ACC2	NDI-010976	Phase 1 (NCT02876796)	↓DNL	^89^
	Fatty acid synthetase (FAS) inhibitor	Orlistat	Phase 4 (NCT00160407)	↓Hepatic steatosis ↓Body weight ↓Insulin resistance ↓ALT, AST ↑Adiponectin	^93^
		TVB-2640	Phase 2 (NCT03938246) Phase 2b (NCT04906421, recruiting)	↓Hepatic steatosis ↓Liver fibrosis ↓Inflammation	^91^
		FT-4101	Phase ½ (NCT04004325, terminated)	↓Hepatic steatosis	^92^
	SCD inhibitor	Aramchol	Phase 3 (NCT04104321, recruiting) Phase 2 (NCT01094158, NCT02279524)	↓Hepatic steatosis ↓ALT, AST, HbA1c	^95,97^
	AMPK activator	PXL770	Phase 1 (NCT03950882)	↑Insulin sensitivity ↓GGT	^593^
	Downregulation of SREBP1-c expression	Oltipraz (OPZ)	Phase 2 (NCT01373554, NCT00956098) Phase 3 (NCT02068339, NCT04142749, recruiting)	↓Liver fat content ↓TGF-beta1	^109,110^
	HMG-CoA reductase inhibitor	Atorvastatin	Phase 2 (NCT02633956) (NCT04679376, recruiting)	↓Hepatic steatosis ↓Serum advanced glycation endproducts	^120,594^
	LXR and SREBP-1c inhibitor	DUR-928	Phase 1b	↓Hepatic steatosis ↓ALT, AST, GGT ↓TC, LDL	^595^
Lipid metabolism	11β-hydroxysteroid dehydrogenase type 1 (11β-HSD1/HSD11B1) inhibitor	RO5093151	Phase 1b (NCT01277094)	↓Liver fat content ↓Body weight ↓ALT, GGT, TC	^596^
Lipid metabolism	Hydrophilic, non-toxic, secondary bile acid in humans	Ursodeoxycholic acid (UDCA)	Phase 4 (NCT04977661)	↓Hepatic steatosis ↓Body weight ↓ALT, AST, LDL, TG, TC ↓Inflammation	^597,598^
	Steroidal agonist of FXR	Obeticholic acid	Phase 3 (NCT02548351) (EudraCT, 20150-025601-6)) Phase 2b (NCT01265498)	↓Hepatic steatosis ↓Liver fibrosis ↓ALT, AST, GGT	^202,203,599^
		EDP-305	Phase 2 (NCT03421431)	↓Liver fat content ↓ALT	^600^
	Non-steroidal agonist of FXR	Tropifexor (LJN-452)	Phase 2b (NCT03517540, NCT02855164)	↓Liver fat content ↓ALT, GGT	^599,601,602^
		EYP001	Phase 2 (NCT03812029)	No results posted	^603^
		Nidufexor (LMB763)	Phase 2 (NCT02913105, NCT03804879)	No results posted	^604^
		Cilofexor (GS-9674)	Phase 2 (NCT02781584, NCT02854605)	↓Hepatic steatosis ↓serum bile acids ↓GGT	^205^
	THR-β agonist	Resmetirom (MGL-3196)	Phase 3 (NCT03900429 EudraCT Number: 2018-004012-22) Phase 2 (NCT02912260)	↓Hepatic steatosis ↓Liver fibrosis ↓LDL, APOB, TG	^218,219^
		VK2809	Phase 2 (NCT02927184 NCT04173065, recruiting)	↓Liver fat ↓LDL	
Lipid metabolism	FGF19 analog	Aldafermin (NGM282)	Phase 2 (NCT02443116, NCT02704364 NCT03912532)	↓Hepatic steatosis ↓ALT, AST, bile acids ↓PRO-C3	^212–214^
Lipid metabolism	Fibroblast growth factor 21 (FGF21) receptor agonist	Pegbelfermin (BMS-986036)	Phase 2 and 2b (NCT03486899, NCT03486912, NCT02413372, NCT02097277)	↓Hepatic steatosis ↓serum PRO-C3 ↑adiponectin ↑HDL, ↓TG	^227,228,605^
		Efruxifermin	Phase 2a (NCT03976401)	↓Hepatic steatosis ↓serum PRO-C3 ↓ALT, AST, GGT, ALP	^229^

**Table 2:** Antidiabetic and lipid metabolism drugs in the preclinical stage

Class	Targets	Drug name	Experimental models	Outcome	Ref.
Antidiabetic drugs	PPARα agonist	Bezafibrate	MCD diet-fed male KK-Ay/TaJcl (KK-Ay) mouse model	↓Lipid accumulation ↓Hepatic inflammation ↓Fibrosis ↓Plasma ALT, TG ↑Hepatic fatty acid β-oxidative genes Bezafibrate reduced the mRNA levels of profibrogenic and fibrogenic genes in TGF-β1-stimulated RI-T cells	^147,149^
		GW7647	Choline-deficient L-amino acid–defined diet containing 45% fat (HF-CDAA) diet-fed mouse model	↓Liver/BW ratio ↓Serum TG ↓Liver steatosis	^148^
		Gemcabene	STAM™ murine model of NASH	↓Hepatic mRNA markers of inflammation, lipogenesis and lipid modulation, fibrosis	^150^
	PPARδ agonist	Seladelpar (MBX-8025)	Atherogenic diet-fed Alms1 mutant (*foz/foz*) mouse model	↓Hyperglycemia, hyperinsulinemia, and whole-body insulin resistance ↓Blood glucose, ALT ↓Apoptosis ↓Inflammation ↓NAS	^151^
Antidiabetic drugs	GLP agonist	Dulaglutide (LY2189265)	HFHC diet-fed mouse model	↓Body weight ↓AST, glucose levels ↓Inflammation No effects on NAS Score and liver TG.	^606,607^
	GLP-1/GLP-2R dual agonist	GLP-1/2-Fc fusion	Choline-deficient high-fat diet with high fructose and sucrose water (CDHF-FC)-fed mouse model	↓Body weight, glucose levels, hepatic TG, and cellular apoptosis. ↓Liver fibrosis, insulin sensitivity.	^608^
	GCGR and GLP-1R dual agonist	Oxyntomodulin (OXM) analog	Diabetogenic diet-induced obese (DIO) mouse model	↓Liver lipid content ↓Fat mass	^609^
Lipid metabolism	ACC inhibitor	ND-630	HFD or HSD diet-rat model	↓Hepatic steatosis ↑Insulin sensitivity ↓Weight gain	^610^
		ND-654	Diethylnitrosamine (DEN) induced HCC rat model	↓Hepatic DNL ↓Development of HCC	^611^
		WZ66	HFD-diet mouse model	↓Seatosis ↓KCs and HSCs activation ↓Hepatic TGs and other lipids including diglycerides (DGs), phosphatidylcholine (PC), and sphingomyelin (SM) ↓*Allobaculum*, *Mucispirillum*, and *Prevotella genera* as well as *Mucispirillum schaedleri* species in gut microbiota.	^612^
	ALOX12-ACC inhibitor	IMA-1	High-fat/high-cholesterol (HFHC) diet-induced NASH mouse model HFHC diet-induced NASH *Cynomolgus* macaque model	↓NASH progression	^613^
Lipid metabolism	Hepatic stimulator substance (HSS)	Overexpression of HSS	HFD or MCD diet-fed HSS gene-transfected mouse mode	↓Hepatic steatosis ↓Hepatic inflammation ↑Activity of CPT-1	^614^
	ACLY inhibitor	Hydroxy citrate	HFD-fed rat model	↓ALT, AST ↓GGT, LDH	^82^
		Bempedoic acid (ETC-1002)	HFD-fed mouse model	↓Body weight ↑Glycemic control ↓Hepatic TG and TC ↓Inflammatory, fibrosis ↓NAS score	^79^
Lipid metabolism	SREBP inhibitor	25-HL	Western-type diet (WD)-fed mouse model Amylin liver NASH model (AMLN) diet-fed *Ldlr*−/− male mice mouse model	↑Energy expenditure ↓TC and TG in serum and liver ↓Hepatic steatosis, inflammation, and fibrosis	^615^
Lipid metabolism (target on adipocytes)	Adipocytes targets	Leukemia inhibitory factor (LIF)	HFD-fed *Adipoq-Cre*; *Lifr*^*fl/fl*^ mouse model	↓Hepatic TG	^616^
Lipid metabolism (target on bile acids)	FXR agonists	Tiliamsoine	HFD-fed, DEN-induced nonalcoholic steatohepatitis rat model	↓Plasma levels of transaminases, phosphatase, and LDH ↓TNFα	^617^
		GC-1 (sobetirome)	Choline-devoid methionine-deficient (CMD) diet-fed rat model	↓Hepatic steatosis	^222,618^
		MGL-3196 (resmetirom)	HFD-fed rat model	↓TC, LDL-C	^220^
Lipid metabolism	FGF21 agonist	EPB-53	HFD-fed mouse model	↓Body weight ↓Glucose tolerance ↓Hepatic steatosis ↓Hypertriglyceridaemia	^619^
Lipid metabolism	P53 agonist	Doxorubicin	HFD-fed mouse model MCD diet-fed mouse model	↓Liver steatosis ↓Lipogenesis, inflammation, and ER stress ↓Liver damage	^329^

##### Targeting de novo lipogenesis (DNL) and hepatic lipid metabolism

DNL can be upregulated by 20–30% in patients with NAFL/NASH compared to healthy controls, and increased lipogenesis is the key feature associated with fatty liver.^73^ DNL refers to the endogenous synthesis of lipids from dietary sources (usually carbohydrates or stored energy depots), including fatty acid synthesis, fatty acid elongation/unsaturation, and assembly into triglycerides (TG).^74^ DNL is mainly regulated by two key transcription factors: sterol regulatory element-binding protein 1c (SREBP-1c, activated by insulin) and carbohydrate regulatory element-binding protein (ChREBP, activated by elevated glucose).^75^ There are also two crucial enzymes that regulate DNL: acetyl-CoA carboxylase (ACC) and fatty acid synthase (FAS). ACC introduces a carboxyl group into acetyl-CoA to produce malonyl-CoA, and FAS is responsible for converting malonyl-CoA to fatty acid chains. DNL is closely associated with excessive glucose intake and the development of IR, which further contributes to the development of NAFL/NASH.

ACLY inhibition: ATP-citrate lyase (ACLY) is a cytoplasmic enzyme responsible for the generation of acetyl-coenzyme A (acetyl-CoA) in DNL and cholesterol synthesis.^76^ The gene expression of ACLY increased both in patients with NAFLD^77^ and leptin receptor-deficient *db/db* mice.^78^ Bempedoic acid (ETC-1002), an ACLY inhibitor, alleviated high-fat diet (HFD)-induced NASH in male C57BL6/N mice, including decreased body weight gain, improved glycemic control, reduced hepatic TG and total cholesterol (TC), lowered mRNA expressions of inflammatory and fibrotic genes (*Ccl2, Timp1*, and *Col1a1*), and improvement in NAS score.^79^ In a phase 3 clinical trial (NCT02666664), Bempedoic acid significantly reduced low-density lipoprotein cholesterol (LDL-C) levels.^80^ Bempedoic acid was recently approved by US FDA for the treatment of heterozygous familial hypercholesterolemia (HeFH)^81^ and clinical atherosclerotic cardiovascular disease (ASCVD), due to the major risk factors for HeFH and ASCVD are the elevated LDL-C levels.^79^ In addition, hydroxy citric acid, another competitive inhibitor of ACLY, significantly reduced fatty acid synthesis and the levels of liver injury parameters, including alanine transaminase (ALT), aspartate transaminase (AST), gamma-glutamyltransferase (GGT), and lactate dehydrogenase (LDH) in rats fed HFD.^82,83^

ACC inhibition: ACC converts acetyl-CoA to malonyl-CoA and is a rate-limiting step in DNL. A preclinical study demonstrated that inhibition of ACC reduced liver fibrosis in a rat choline-deficient, HFD model.^84^ In a randomized and placebo-controlled trial of patients with NASH (NCT02856555), median relative decreases in magnetic resonance imaging-estimated proton density fat fraction (MRI-PDFF) were greater in patients treated with 20 mg of ACC inhibitor GS-0976 for 12 weeks (decrease of 29%) than those given placebo (decrease of 8%; *p* = 0.002).^85^ GS-0976 decreased hepatic steatosis, selected fibrosis markers, and liver biochemistry.^85^ The clinical study also indicated that after administration of GS-0976 for 12 weeks, the median hepatic DNL was decreased by 22% from baseline in patients with NASH (*p* = 0.004).^86^ MK-4074 is a small-molecule inhibitor specifically targeting liver ACC1/2. In preclinical animal models and clinical studies, administration of MK-4074 showed suppressed DNL and enhanced liver fatty acid oxidation (FAO), leading to significantly reduced hepatic TG content in preclinical studies.^87^ Based on the promising results from the pilot studies, phase 1 clinical studies have been conducted to assess changes in liver fat content (NCT01431521) in adult men and women with NAFL after multiple oral doses of MK-4074 and Pioglitazone hydrochloride. The results showed that the administration of MK-4074 for 1 month reduced liver TG by 36% in patients with hepatic steatosis. However, although liver TG content was reduced, plasma TG significantly increased by 200%.^87^ Similarly, dose-dependent reduction in liver fat reached 50–65% and a dose-dependent elevation in serum TG reached 8% with the ACC inhibitor PF-05221304 (NCT03248882). Notably, PF-05221304 combined with PF-06865571 (Diacylglycerol O-Acyltransferase 2 (DGAT2) inhibitor) has the potential to avoid some limitations of ACC inhibitor alone, including the ACC inhibitor-mediated elevation in serum TG (NCT03776175).^88^ In addition, an allosteric inhibitor of ACC1/2, NDI-010976, was well tolerated at doses up to 200 mg and resulted dose-dependently in inhibition of hepatic DNL in obese adult male subjects (NCT02876796).^89^

FAS inhibition: FAS is a rate-controlling enzyme that converts malonyl-CoA to palmitic acid during DNL. FAS mRNA expression in the liver is significantly higher in patients with NAFL/NASH than that in normal subjects.^90^ In a phase 2 clinical trial, administration of TVB-2640, a FAS inhibitor, showed promising results in adult patients with NASH with ≥8% liver fat and liver fibrosis (NCT03938246).^91^ TVB-2640 reduced liver fat by 9.6% in the 25 mg cohort and 28.1% in the 50 mg cohort from baseline compared to 4.5% increase in liver fat in the placebo cohort. A total of 23% of patients in 25 mg group and 61% in 50 mg group of TVB-2640 achieved a relative reduction of liver fat of ≥30% respectively, while only 11% of patients in the placebo group.^91^ A phase 2b (NCT04906421) trial is recruiting and subjects with liver fibrosis at stages F2–F3 will be enrolled to further evaluate the safety and efficacy of TVB-2640 in subjects with NASH. The FAS inhibitor (FT-4101) safely reduced hepatic DNL and steatosis of patients with NASH in a phase 1/2 clinical trial (NCT04004325).^92^ Another FAS inhibitor Orlistat, however, did not enhance weight loss or improve liver enzymes, measures of IR, and histopathology (NCT00160407).^93^

SCD1 inhibition: stearoyl coenzyme A desaturase 1 (SCD1) is an enzyme that catalyzes the rate-limiting step in the formation of MUFAs, specifically oleate and palmitoleate from stearoyl-CoA and palmitoyl-CoA.^69,70^ The expression of SCD1 in the liver was increased both in patients with NAFLD and *ob/ob* mice.^94^ Aramchol is a conjugate of cholic acid and arachidic acid that had an inhibitory effect on SCD1 activity to reduce liver fat content in patients with NASH (NCT01094158).^95^ In both isolated primary human hepatic stellate cells (HSCs) and a human hepatic stellate cell line (LX-2), Aramchol reduced fibrogenic gene expression by inhibiting SCD1 and inducing PPARγ.^96^ In a phase 2 clinical trial (NCT01094158) for NASH, liver fat content was decreased by 12.57% in patients treated with 300 mg/day of Aramchol compared with the increase of 6.39% in the placebo group.^95^ In a 52-week, double-blind, placebo-controlled, phase 2b trial (NCT02279524), Aramchol displayed a placebo-corrected decrease in liver TG, without reaching the prespecified significance (*p* = 0.066). NASH resolution without worsening fibrosis was achieved in 16.7% of Aramchol vs. 5% of the placebo, and fibrosis improvement by ≥1 stage without worsening NASH in 29.5% vs. 17.5%, respectively.^97^ Despite administration of 600 mg of Aramchol was unable to reduce liver fat, safety and changes in liver histology and enzyme improvements were observed; therefore, Aramchol is processing into a phase 3 trial (NCT04104321).^97^

SREBP inhibition: SREBP-1c is an insulin-sensitive transcription factor that plays a key role in the induction of lipogenic genes in the liver, which is transactivated by liver X receptor (LXR).^98^ The increased levels of nuclear SREBP-1c contributed to the hepatic steatosis^99^ and were regulated by insulin in mouse or rat T2DM models.^100^ Nuclear accumulation of mature forms of SREBP-1c and expression of its target genes is blocked by the mechanistic target of rapamycin kinase (mTOR) complex 1 (mTORC1) inhibitor rapamycin.^101^ The protein folliculin (FLCN) in adipocytes phosphorylates mTOR and retains TFE3 in the cytoplasm to inhibit WAT browning.^102^ This process is independent of canonical mTOR-S6K signaling.^102^ A recent study has shown that deletion of *Flcn* in the liver inhibited mTORC1 signaling to promote nuclear translocation of TFE3, which in turn activated lipid catabolism genes and suppressed DNL genes.^103^ This specific deletion of hepatic *Flcn* inhibited the activation of SREBP-1c and could prevent or reverse NASH in mice fed choline-deficient L-amino acid–defined and high-fat (CDAA-HF) diet.^103^ Previous reports have shown that hyperactivation of SREBP-1c promotes hepatic TG accumulation,^104,105^ suggesting that targeting SREBP-1c for regulating hepatic lipid metabolism might be an appropriate strategy for NASH treatment.^105,106^ Oltipraz (OPZ) is a synthetic dithiolethione with an antisteatotic effect by inhibiting the activity of LXR-α, thereby suppressing SREBP-1c activity.^107^ Administration of the thiol-reactive agent OPZ significantly attenuated the progression of histologic abnormalities, especially hepatic fibrosis in rats on a CDAA diet.^108^ The efficacy and safety of OPZ administration in patients with NAFLD were verified in the phase 2 clinical trials (NCT01373554 and NCT00956098). NCT01373554 revealed that 24-week treatment of OPZ significantly reduced the liver fat content in a dose-dependent manner in patients with NAFLD. Compared with the placebo group (–3.2%), absolute changes in the liver fat content were reduced by 7.7% and 13.9% for the low-dose and high-dose groups (*p* = 0.13 and *p* < 0.01), respectively.^109,110^ Clinical phase 3 trials (NCT04142749 and NCT02068339) are carried out to investigate the inhibitory role of OPZ on fatty acid synthesis in patients with NAFLD.

SREBP2 transcriptionally controls 3-hydroxy-3-methyglutaryl-coenzyme A (HMG-CoA) reductase, which is a key enzyme in cholesterol synthesis and ketogenesis that closely links to the development of NAFL/NASH.^111,112^ Statins (HMG-CoA reductase inhibitors) restrict cholesterol synthesis and are mostly used as hypolipidemic drugs. It has been shown that Statins increased the FAO capacity of the liver by inducing PPARα and prevented the development of MCD-induced NASH in mice; however, the authors did not claim that the effect of Statins on improving NASH may not be related to its cholesterol decreasing function.^113^ Simvastatin, first-generation statins, treatment in vivo or in vitro inhibited the activation of HSC in rats fed HFD.^114^ In addition, Atorvastatin, a third-generation synthetic statin that is more effective to reduce cholesterol and LDL-C, dissolved cholesterol crystals (the focal point of the coronal structure of activated Küpffer cells (KCs)) to improve fibrosis in obese and diabetic *Alms1* mutant (*foz/foz*) mice fed high-fat (23%) diet containing 0.2% cholesterol.^115^ In a 6-year follow-up of more than 11 million subjects, Statins were observed to reduce the risk of NAFLD (adjusted odds ratio (AOR) 0.66; 95% confidence interval (CI) 0.65–0.67) and to reduce the risk of liver fibrosis (AOR 0.43; 95% CI 0.42–0.44).^116^ A case–control study showed a protective effect of Statins against NAFLD-associated HCC (OR = 0.20, 95% CI: 0.07–0.60, *p* = 0.004) in 102 subjects (patients with NAFLD-associated HCC, *n* = 34; NAFLD patients without HCC, *n* = 68).^117^ Probably due to the hepatotoxic effects of Statins, doctors may not prescribe statins to patients with high plasma aminotransferase levels.^118^ However, a post-hoc analysis of Statins use in a randomized controlled trial revealed that Statins therapy was safe for patients with prediabetes or T2DM and NASH, suggesting Statins may be a potential therapeutic strategy in those patients.^119^ Patients with NAFL/NASH have a higher risk of CVD.^38^ Further investigation is needed to fully demonstrate the safety and efficacy of statins, doctors may be able to try to use statins in patients with NASH to reduce the risk of CVD complications. Furthermore, in a phase 2, double-blind, randomized, placebo-controlled, multicenter study (NCT02633956) that evaluated the effect of Obeticholic acid (OCA, a synthetic bile acid (BA), and farnesoid X receptor (FXR) agonist), and the subsequent addition of Atorvastatin therapy, on lipoprotein metabolism in subjects with NASH (fibrosis stages 1–4), OCA-induced increases in LDL-C in patients were mitigated with Atorvastatin.^120^

##### Antidiabetic drugs for NASH treatment

NAFL/NASH is a metabolic-related liver disease with a bidirectional and significant relationship with obesity and T2DM.^121^ In patients with T2DM, the global prevalence of NAFL/NASH is more than 55%.^33^ T2DM has also been linked to a faster progression of NASH, cirrhosis, or HCC.^122^ Although there is no approved drug for the treatment of NAFL/NASH, various antidiabetic agents showed some efficacy. Here we discuss the putative molecular mechanisms that potentially link NAFLD and T2DM, as well as the current pharmacological treatments for NAFLD patients with a metabolic disorder.

PPAR signaling: peroxisome proliferator-activated receptors (PPARs) are ligand-activated transcription factors belonging to the nuclear hormone receptor superfamily. PPARs have three identified isotypes (α, β/δ, and γ), all of which are involved in lipid metabolism and glucose homeostasis in NAFL/NASH. PPARα gene expression in the liver of obese patients negatively correlated with steatosis, NASH severity, and IR.^123^ PPARβ/δ mRNA expression level was reduced in liver biopsies of patients with moderate or severe steatosis.^124^

PPARα is the primary regulator of hepatic fat catabolism during fasting.^125^ It has been demonstrated that deletion of PPARα promoted NAFL/NASH and hepatic inflammation in mice.^126^ It was observed that lipid accumulated massively in the liver of global PPARα knockout (*Ppara*^*−/−*^) mice fed with HFD.^127^ Hepatic steatosis was reduced in hepatocyte-specific PPARα deficient mice compared to *Ppara*^*−/−*^ mice, probably due to increased FAO in other extrahepatic tissues like brown adipose tissue, muscle, and heart.^128^

Diabetic mice with hepatocyte-specific deletion of PPARγ had improved hepatic steatosis yet more severe IR, probably due to reduced insulin sensitivity in muscle and adipose tissues.^129^ PPARγ agonists reduced hepatic steatosis in patients with NAFLD possibly due to effects in adipose tissue, where PPARγ activation promoted adipogenesis in adipose tissue to decrease the fatty acids entering the liver.^130^ In addition, deletion of PPARγ in non-parenchymal liver cells including KCs and HSCs exacerbated liver damage and fibrogenic response to carbon tetrachloride (CCl_4_) challenge.^131^

The expression levels of PPARβ/δ are generally higher than PPARα and PPARγ. PPARβ/δ plays a critical role in the liver, skeletal muscle, adipose tissue and immune system.^132^ Transcriptional profiling of liver tissue revealed that PPARβ/δ deletion downregulated pathways including lipoprotein metabolism and glucose utilization, and upregulated genes connected to innate immunity and inflammation, which collectively correlated with increased plasma glucose and TG.^133^ PPARβ/δ-deficient mice with HFD were more prone to obesity.^134^ Activated PPARβ/δ suppresses hepatic glucose output and promotes beta-oxidation in muscle to regulate metabolic homeostasis.^135^ Meanwhile, fatty acid uptake by skeletal muscle appears to be influenced by hepatic PPARβ/δ, with hepatocyte-specific PPARβ/δ deficiency reducing muscle fatty acids uptake to avoid lipotoxicity in hepatocytes.^136^ In addition, PPARs are involved in anti-inflammatory effects through a mechanism known as transrepression that inhibits nuclear factor-κB (NF-κB), activator protein-1 (AP-1), signal transducer and activator of transcription (STAT), or nuclear factor of activated T cells.^137^

In general, PPARs are involved in glucose and lipid metabolism in multiple organs and contribute to the anti-inflammatory response in NAFL/NASH. Currently, there are several drug candidates for PPAR activations, which will be described later. In addition, compared to selective PPAR agonists, targeting two isotypes or pan-PPAR agonists combines the beneficial effects of selective PPAR agonists and improves NAFL/NASH more effectively in multiple ways.^138,139^

PPAR agonists: a variety of agents targeting different subtypes of PPAR are currently under preclinical and clinical studies. Pioglitazone, a promising PPARγ agonist, is currently under a phase 4 clinical trial (NCT00994682). The study included 44% of patients with type 2 diabetes who have NASH regression and 26% of non-diabetic patients who have NASH regression. A significant regression in fibrosis was observed only in patients with type 2 diabetes (*p* = 0.035). Compared with non-diabetic patients, Pioglitazone significantly improved insulin sensitivity in adipose tissue of diabetic patients (*p* < 0.001).^140^ However, compared to patients with prediabetes, Pioglitazone reduced liver fibrosis and adipose tissue insulin sensitivity at significantly higher levels in patients with type 2 diabetes.^140,141^ It is demonstrated that Elafibranor (GFT505), a dual PPARα/δ agonist, had liver-protective effects on steatosis, inflammation, and fibrosis in several animal models of NAFL/NASH.^142,143^ In a phase 2b clinical trial (NCT01694849), compared with the placebo group, NASH resolved without fibrosis worsening in a higher proportion of patients in the 120-mg Elafibranor group vs. the placebo group (19% vs. 12%; *p* = 0.045).^143^ However, Elafibranor exhibited poor anti-NASH effects in a phase 3 clinical trial (NCT02704403), probably due to its weak PPARα/δ agonistic activity and poor metabolic stability. Novel, structurally stable PPARα/δ agonists are still under investigation, such as new Triazolone derivatives.^144^ Other promising PPAR agonists were evaluated in randomized controlled phase 2/3 trials of NASH patients including the dual PPARα/γ agonist Saroglitazar (NCT03061721)^145^ and a pan-PPAR agonist Lanifibranor (NCT03008070).^146^ In a clinical 2b trial (NCT03008070), Lanifibranor achieved optimal results that patients treated with the 1200-mg dose of Lanifibranor had a decrease of at least two points in the SAF-A score (the activity part of the Steatosis, Activity, Fibrosis (SAF) scoring system) without worsening of fibrosis and reached the primary endpoint, which will ultimately determine the therapeutic potential of pan-PPAR agonist targeting inflammation and fibrosis to support further evaluation of Lanifibranor in the phase 3 trial.^146^ In addition, the study also reached several primary secondary endpoints, including NASH remission, no deterioration of NASH, and improved liver fibrosis. Furthermore, in preclinical studies, multiple PPAR agonists have achieved antifibrotic results in both animal and in vitro models, such as PPARα agonists (Bezafibrate^147^ and GW7647^148^).^149^ PPARα agonist Gemcabene prevented steatosis, inflammation, and hepatocyte ballooning, and inhibited fibrosis progression in a high-fat/high-calorie diet-fed murine model of NASH.^150^ PPAR δ agonist Seladelpar (MBX-8025) reduced steatosis and liver inflammation, and improved liver fibrosis in diabetic obse mice.^151^

While preclinical/clinical studies suggest that the dual/pan-PPAR agonists have a more significant effect in the treatment of disease when compared with PPARα or PPARγ agonists alone, improvement of the low agonistic activity and low metabolic stability of multiple agonists may still need to be improved. In addition, it is important to note that side effects occur frequently, such as diarrhea, nausea, peripheral edema, anemia, and weight gain.

GLP-1 agonists: glucagon-like peptide-1 (GLP-1), a secreted peptide from enteroendocrine L cells, promotes insulin secretion and β-cell proliferation in the pancreas and regulates blood glucose levels.^152^ Interestingly, GLP-1 levels are decreased in NAFL/NASH patients.^153^ Liraglutide, a GLP-1 analog, is used as an antidiabetic agent by induction of insulin secretion. In a double-blind, randomized, placebo-controlled phase 2 study (NCT01237119), 39% of patients with NASH treated with Liraglutide showed NASH regression compared with 9% of patients in the placebo group. In addition, 9% of patients in the Liraglutide group developed fibrosis, while 36% of patients in the placebo group developed fibrosis. These observations indicate that Liraglutide is a well-tolerated disease-modifying intervention leading to histological resolution of NASH,^154,155^ reducing metabolic dysfunction, IR, and lipotoxicity in the pathogenesis of NASH.^156^ Another phase 2 study (NCT01399645) was conducted to test the effects of insulin vs. Liraglutide therapy on hepatic fat in patients with T2DM inadequately controlled with Metformin therapy. However, Liraglutide treatment did not significantly alter the liver mean proton density fat fraction (PDFF) (*p* = 0.15), magnetic resonance spectroscopy-PDFF (*p* = 0.80), liver volume (*p* = 0.30), or the total liver fat index (*p* = 0.39).^157^ Semaglutide, a GLP-1 receptor agonists (GLP-1-Ra) developed based on the extensive research behind the development of Liraglutide, has been used to treat T2DM. In a randomized, double-blind, placebo and active controlled phase 2 trial (NCT02453711), Semaglutide showed clinically relevant weight loss compared with placebo at all doses.^158,159^ Patients treated with 0.4 mg of Semaglutide had improvements in fibrosis stage compared with patients in the placebo group (43% vs. 33%) in a phase 2 clinical trial (NCT02970942).^160–162^ Given the potent effects of Semaglutide, several clinical trials are underway to determine whether Semaglutide alone or in combination with other drugs could better benefit patients with NASH (NCT04822181, NCT05016882, NCT04971785, NCT05195944, NCT04639414, and NCT04944992). Recently, a dual glucose-dependent insulinotropic polypeptide (GIP)/GLP-1 receptor agonist Tirzepatide (LY3298176) significantly reduced NASH and fibrosis biomarkers in patients with T2DM.^163,164^ Another dual GIP/GLP-1 receptor agonist NNC0090-2746 improved glycemic control and reduced body weight and TC.^165^ In addition, a balanced glucagon-GLP-1 receptor agonist (Cotadutide) was observed to improve lipid profile, hepatic function indexes, and NAFLD fibrosis markers in type 2 diabetes patients controlled with Metformin.^166^

The most common adverse events of GLP agonists for NASH are mild to moderate gastrointestinal side effects, including nausea, diarrhea, indigestion, and vomiting, which show in a dose-dependent manner and are often transient.^167^ In addition to the most frequently observed gastrointestinal side effects, Liraglutide raised serum lipase and amylase levels. The absolute risk of Liraglutide-induced acute pancreatitis was higher when compared to placebo. Liraglutide may also contribute to an increased risk of acute gallbladder or biliary disease.^168,169^

SGLT2 inhibition: sodium-glucose cotransport protein 2 (SGLT2) inhibitors are a relatively new class of antidiabetic agents that lower blood glucose by inhibiting glucose reabsorption by SGLT2 in the proximal renal tubules.^170^ A growing number of studies have shown that most SGLT2 inhibitors are effective in improving steatosis and fibrosis in patients with NAFL/NASH and T2DM.^171–174^ In addition, SGLT2 inhibitors can also block KCs activation and associated inflammatory processes.^175^ Results of clinical trials (UMIN000015727 and jRCTs071180069) indicated that long-term Ipragliflozin treatment (IPR group) ameliorated hepatic fibrosis in patients with NAFL/NASH. It was reported that 67% of the IPR group (50 mg/day for 72 weeks) were relieved from NASH compared to 27.3% in the control group. In addition, none of the participants in the IPR group developed NASH, whereas 33.3% of the control group developed NASH. Compared to baseline measurements in patients with NASH, body weight, hemoglobin A1c (HbA1c), hepatic function indexes (AST, ALT, and GGT), body fat mass, and steatosis were significantly decreased after Ipragliflozin oral administration (50 mg/day) for 24 weeks.^176,177^ Empagliflozin, another SGLT2 inhibitor was analyzed in several clinical trials (Institutional Review Board of NAMS (approval number: 547-077/078),^178^ NCT02964715, IRCT20190122042450N3).^179^ After Empagliflozin administration for 6 months, there was a significant reduction in the mean controlled attenuation parameter (CAP) value from 282.07 ± 47.29 to 263.07 ± 49.93 dB/m and liver stiffness (LS) from 5.89 ± 4.23 to 5.04 ± 1.49 kPa.^178^ Empagliflozin (25 mg daily for 24 weeks) improved steatosis (67% vs. 26%, *p* = 0.025), ballooning (78% vs. 34%, *p* = 0.024), and fibrosis (44% vs. 6%, *p* = 0.008) significantly compared with historical placebo.^180^ Study also showed that CAP score significantly decreased in borderline with Empagliflozin (10 mg for 24 weeks) compared to placebo.^179^ These data suggest that long-term Empagliflozin treatment has improved liver steatosis and fibrosis in patients with NAFL/NASH and T2DM, leading to beneficial effects, such as weight loss and reduction in hepatic fat, transaminases, and GGT content. Canagliflozin, an SGLT2 inhibitor, significantly improved several hepatic functions or fibrosis markers (AST, fibrosis-4 index, and FM-fibro index), and metabolic parameters (HbA1c and body weight).^181^ Canagliflozin may be useful for the treatment of T2DM patients with NASH, especially those patients in hepatic fibrosis stages 1–3 (UMIN000023044).^181^ There is a reduction in visceral fat and an improvement in liver tests, including serum concentrations of AST, ALT, ferritin, and type IV collagen 7S, after treatment with Dapagliflozin by inhibiting SGLT2 (UMIN000022155 and UMIN000023574).^182,183^ However, administration of Dapagliflozin for 12 weeks did not improve hepatic steatosis in patients without T2DM (NCT02696941).^184^

##### Bile acids (BAs) therapeutics

BAs promote the intestinal absorption of lipid substances and improve lipid hydrolysis metabolism through regulating various lipid metabolism enzymes and enhance the lipid metabolism of the pancreas.^185^ The level of total fecal BAs was elevated in patients with NAFL/NASH, suggesting that the progression of NAFL/NASH might be associated with altered BAs homeostasis.^186^ Administration of cholic acid, a primary BA, changed the bacterial composition of the intestinal microbiome,^187^ and the increase of circulating BAs led to the toxic accumulation of BAs in hepatocytes, which propagates inflammation, oxidative stress, and the worsening of NAFL/NASH.^188–190^ FXR signaling is activated by BAs and the most potent of which is chenodeoxycholic acid (CDCA).^191,192^ FXR signaling was inhibited in patients with NAFLD and rats fed an HFD, probably due to deoxycholic acid (DCA), an FXR antagonistic secondary BA, increased while the agonistic CDCA was decreased.^193^ In the light of the potential hepatotoxic effects of BAs and BAs-induced FXR signaling to regulate insulin sensitivity and glycolipid metabolism, raising attention was attracted in the role of BAs in the treatment of NAFL/NASH (Tables 1 and 2). For instance, ursodeoxycholic acid (UDCA) is a hydrophilic, non-toxic, secondary BA in humans. In a phase 4 clinical trial (NCT04977661), UDCA improved hepatic aminotransferases and serum cytokine and chemokine (41%, 35%, 47%, and 37% for ALT, AST, IL-6, and CCL2/MCP-1, respectively).^189^

FXR agonists: FXR is found mainly in the liver and intestine, a major intercellular BA receptor activated during the fed state to regulate metabolism and inflammation.^185,194,195^ The interaction of BAs and intracellular FXR not only inversely regulates BA synthesis, but inhibits hepatic adipogenesis and steatosis, reduces hepatic gluconeogenesis, and increases peripheral insulin sensitivity through transcription of GLUT4.^185,196–198^ Deficiency of FXR leads to increased BA synthesis, which further contributes to liver fibrosis and inflammation and even to HCC.^199–201^

OCA, an FXR agonist, regulates the expression of transcription factors that reduce BA synthesis and liver steatosis.^202^ In a clinical phase 2 trial (NCT01265498), OCA improved the histological features of NASH. A total of 45% of patients in the OCA group had improved liver histology compared with 21% of patients in the placebo group (*p* = 0.0002).^203^ Furthermore, in an 18-month clinical phase 3 trial (NCT02548351), 23% of the patient cohort who received OCA achieved a reduction of NAS by at least one score without worsening fibrosis compared to 12% (37/311) in the placebo group, indicating that OCA improved inflammation and fibrosis in patients with NASH.^202^ In addition, several other FXR agonists are currently under phase 2/3 trials, including Cilofexor, Tropifexor, and Nidufexor.^204^ In a recent phase 2b study (NCT02854605), Cilofexor (GS-9674) improved hepatic steatosis and liver transaminase in NASH patients. The relative decrease of MRI-PDFF in patients treated with 100 mg of Cilofexor for 24 weeks was 22.7%, while that of patients treated with placebo increased by 1.9% (*p* = 0.003). A total of 39% of the patients with Cilofexor treatment (*p* = 0.011) and 13% of the patients in the placebo group showed ≥30% reduction in MRI-PDFF (NCT02854605).^205^ A double-blind phase 2 study proved that EDP-305, a non-BA FXR agonist, mildly reduced ALT levels and liver fat content (NCT03421431). The mean reductions from baseline in ALT for patients receiving 2.5 and 1 mg of EDP-305 for 12 weeks were 27.9 U/L (*p* = 0.049) and 21.7 U/L (*p* = 0.304), respectively, compared to a decrease of 15.4 U/L for those receiving placebo. Absolute liver fat reduction was 7.1% with 2.5 mg EDP-305, 3.3% with EDP-305 1 mg, and 2.4% with placebo.^206^ While FXR agonists displayed promising efficacy in treating patients with NASH, almost all FXR agonists caused side reactions, such as pruritus and deterioration of the high-density lipoprotein (HDL-C)/LDL-C ratio.^207^

There is a strong association between impaired fibroblast growth factor 19 (FGF19) signaling and elevated levels of BAs in circulation.^208–210^ FGF19 modulates hepatic fat metabolism via multiple mechanisms, including accelerating lipid oxidation and repressing hepatic DNL, subsequently protecting the liver from steatosis.^211^ Aldafermin (NGM282), an engineered analog of the gut hormone FGF19, showed a tendency towards reducing liver fat and improving fibrosis yet with adverse events, including diarrhea, abdominal pain, and nausea in NASH patients.^212–214^

THR-β agonists: thyroid hormone receptor beta (THR-β) is the main thyroxine receptor in the liver and mediates cholesterol metabolism and excretion through BAs.^215,216^ THR-β agonists have been observed to reduce lipotoxicity, improve liver function and subsequently reduce liver fat by promoting fatty acid breakdown and stimulating mitochondrial biogenesis.^217^ Resmetirom (MGL-3196) is a selective THR-β agonist and is currently under clinical phase 2/3 trials (NCT02912260 and NCT03900429). In a 36-week paired liver biopsy study (NCT02912260), markers of fibrosis were reduced significantly by Resmetirom treatment, including the reductions in LS (*p* = 0.015) and the ratio of PRO-C3 (N-terminal type III collagen pro-peptide)/C3M (metalloproteinase-degraded collagen III) (*p* = 0.0004), a proposed measure of net fibrosis formation, in adult patients with NASH.^218,219^ Furthermore, the effective and safe daily doses of Resmetirom at 80 and 100 mg were used in the ongoing phase 3 NASH study (NCT03900429).^218^ GC-1 (Sobetirome) and VK2809 are NASH treatment candidates based on THR-β-agonism.^220^ In the human hepatocyte-derived Huh-7 cell line, treatment with GC-1 upregulated the transcription of mitochondrial carnitine palmitoyl transferase 1a (CPT1a), which is part of a mitochondrial outer membrane fatty acid transfer complex, with a dose-response comparable to that of the native THR ligand, triiodothyronine (T3).^220,221^ GC-1 also reduced fat accumulation and improved steatohepatitis induced in rats by a choline-methionine-deficient (CMD) diet.^222^ VK2809 has been shown to reduce the liver fat content in patients with NAFLD after 12 weeks of treatment.^223^

##### Other metabolic pathway targets

Fibroblast growth factor 21 (FGF21) was shown to participate in lipid oxidation and TG clearance in the liver.^224,225^ FGF21 agonists displayed promising effects in preclinical models of NAFL/NASH as well as in short-term clinical trials in patients with NASH.^226^ In a phase 2a study (NCT02413372), 16-week Pegbelfermin (BMS-986036, an FGF21 agonist) administration in patients with NASH and stage 1–3 fibrosis was associated with a significant reduction in hepatic steatosis measured by MRI-PDFF and improvement in lipid profiles, adiponectin concentration, and biomarkers of fibrosis and hepatic injury.^227^ To further evaluate the efficacy of Pegbelfermin, multicenter, double-blind, placebo-controlled, randomized trials (NCT03486899 and NCT03486912) are currently underway to focus on NASH patients with bridging fibrosis and cirrhosis.^228^ Efruxifermin is a long-acting Fc-FGF21 fusion protein designed to mimic the biological activity of FGF21. In a phase 2a clinical trial (NCT03976401), treatment of Efruxifermin in NASH patients (F1-F3 stage) indicated that the absolute changes from baseline in hepatic fat fraction were decreased in a dose-dependent manner, namely –12.3% (28 mg), –13.4% (50 mg), and –14.1% (70 mg) compared to 0.3% in the placebo group.^229^

#### Anti-cellular stress

Chronic disorders of lipid metabolism are closely associated with changes in the redox balance that affect metabolic-associated organelles, resulting in cell lipotoxicity, lipid peroxidation, chronic endoplasmic reticulum (ER) stress, and mitochondrial dysfunction (Figs. 2 and 3).^230^ Excessive accumulation of lipids leads to overproduction of reactive oxygen species (ROS) in different sources, including mitochondria, ER, and NADPH oxidase. Although there is no direct clinical evidence of a clear mechanism of action by which oxidative stress affects NAFLD, oxidative stress markers such as nitric oxide, thiobarbituric acid-reactive species,^231^ and malondialdehyde (MDA)^232^ may be measured clinically to determine the progression of NAFLD. Here we focus on the role of stress in mitochondria and ER in the development of NAFL/NASH.

##### Mitochondrial dysfunction

Energy homeostasis in hepatocytes is mainly mediated by oxidative mitochondrial metabolism, including β-oxidation of free fatty acids (FFAs), tricarboxylic acid (TCA) cycle, ATP synthesis, and ROS production.^233–235^ Wild-type mice exhibited a marked reduction in FAO in liver mitochondria after 4 weeks of HFD feeding, and this effect was restored after 8 weeks,^236^ suggesting a resilient mitochondrial functional change in obesity-induced metabolic disorder. Inefficient β-oxidation of fatty acids leads to the accumulation of toxic lipids such as hepatic diacylglycerols, ceramides, and long-chain acylcarnitines, accelerating inflammation and the NASH process.^237^ During IR, the hepatic TCA cycle decreases mitochondrial respiratory efficiency by increasing electron deposition into inefficient respiratory chains that are prone to generate ROS.^238^ During the development of NAFL/NASH, FFAs overload the mitochondria, FAO, and electron flux in the electron transport chain (ETC) increasing and disrupting mitochondrial homeostasis, leading to excessive production of ROS due to the lack of upregulation of ETC complex activity, which generates “electron leakage” and subsequently exacerbates lipid accumulation in hepatocytes.^239,240^ In addition, the ROS clearance capacity in NAFL/NASH liver is also diminished. For example, glutathione peroxidase (GPx) is one of the most important antioxidant enzymes for maintaining ROS homeostasis; however, in the livers of patients with NASH, GPx activity was greatly reduced.^241^ Manganese (Mn) is mainly responsible for scavenging ROS in mitochondrial oxidative stress, and deficiency or excess of Mn leads to changes in manganese superoxide dismutase activity, resulting in mitochondrial dysfunction.^242,243^ Hydrogen peroxide is mostly catabolized by catalase, an enzyme that catalyzes hydrogen peroxide into molecular oxygen and water without the production of free radicals. In fatty liver, the reduced activity of catalase further promotes the accumulation of ROS.^244^ In addition to ETC, there are other potential sources of ROS in mitochondria, such as mitochondrial flavoenzymes, including pyruvate dehydrogenase, glycerol phosphate dehydrogenase, monoamine oxidase, and α-ketoglutarate dehydrogenase.^235^ Furthermore, an increase in mitochondrial cytochrome P450 2E1 (CYP2E1) expression also leads to increased lipid peroxidation and ROS production and is associated with the progression of NAFL to NASH.^245–247^ The c2 allele of CYP2E1 gives it higher transcriptional and pro-oxidant activity, which determines the susceptibility to develop NASH at the genetic level.^248^

Liver mitochondrial DNA (mtDNA) from patients with NAFL/NASH has a higher rate and degree of heterogeneity of mutations, including mutations in the oxidative phosphorylation (OXPHOS) chain genes.^249^ Mutations in mitochondria encoding cytochrome B, a member of the OXPHOS system, positively correlate with the severity of NAFL/NASH.^250^ Under lipid overload, mtDNA released from damaged hepatocytes acts as danger-associated molecular patterns (DAMPs), inducing upregulation of IL-33 in macrophages via TLR9 receptor, and enhances lipopolysaccharide (LPS)-induced production of IL-1β and TNFα.^251^ Moreover, mtDNA also directly activates HSCs, driving liver fibrosis progression.^252^

##### ER stress

ER is the primary site of lipid synthesis and protein folding and assembly; however, lipid stress, such as lipid overload and impaired VLDL-TG assembly, activates a specific signaling pathway called the unfolded protein response (UPR).^253^ UPR consists of three transmembrane proteins: protein kinase RNA-like ER kinase (PERK),^254^ activating transcription factor 6 (ATF6),^255^ and inositol-requiring signaling protein-1 (IRE1),^256^ which all form stable complexes with the regulatory protein glucose regulatory protein 78 (GRP78, also known as Bip) under normal conditions, while upon ER stress, they dissociate from GRP78 and activate downstream signaling pathways.^257^ Activation of PERK leads to phosphorylation of eukaryotic translation initiation factor-2α (eIF2α) to attenuate global protein translation to reduce the unfolded protein load to ER.^254^ Meanwhile, transcription of activating transcription factor 4 (ATF4) is upregulated^258^ and promotes the transcription of the CCAAT/enhancer binding protein homolog (CHOP), which is a transcription factor associated with apoptosis.^259^ Upon ER stress, ATF6 translocates from the ER to the Golgi where it is cleaved to its active form,^260^ and activated ATF6 stimulates the expression of ER molecular chaperone-related genes. Phosphorylation of IRE1 activates its endoribonuclease activity to splice XBP-1 mRNA, leading to the upregulation of ER chaperones and ER-associated degradation proteins.^261–263^ The initial activation of UPR is to restore ER homeostasis, whereas unresolved ER stress via long-term lipotoxicity promotes apoptosis through the apoptotic signaling pathway downstream of the UPR.

Each disulfide bond formed during protein folding should generate a single ROS,^264^ prolonged ER stress increases UPR-mediated ROS production through activation of CHOP.^265^ In a mouse NASH model, CHOP expression was significantly upregulated.^266^ CHOP deficiency did not improve steatosis but reduced inflammation and apoptosis in NASH mice induced by the MCD diet, indicating CHOP may play a more predominant role in subsequent liver damage by suppression of apoptosis initiation in addition to affecting steatosis.^267^ The transcription factor nuclear factor-E2-related factor-2 (Nrf2) is phosphorylated by ER eIF2 and inhibits lipid accumulation and oxidative stress in the liver by interfering with lipogenic pathways and inducing the expression of antioxidative stress genes.^268^ Deletion of Nrf2 increased oxidative stress, leading to rapid progression of steatosis to NASH in mice fed with an MCD diet.^269,270^

In addition, the ER is a major intracellular calcium storage site, and prolonged exposure to FFAs causes calcium leakage from the ER.^271,272^ Alterations in fatty acids and lipid composition decrease sarco/endoplasmic reticulum calcium ATPase (SERCA) activity, which pumps calcium from the cytoplasm into the ER.^273^ Calcium leaking from the ER may accumulate in the mitochondria, transmitting and amplifying apoptotic signals.^271,272^

##### Antioxidative stress agents

Vitamin E was originally found as a dietary factor preventing fetal resorption and had important effects on reproduction in rats.^274^ Vitamin E has eight natural forms, containing four tocopherols (α-, β-, γ-, and δ-) and tocotrienol (α-, β-, γ-, and δ-). The most abundant of them is -tocopherol, which has strong antioxidant properties.^275^ In addition, the non-antioxidant effects of α-tocopherol, including specific inhibitory effects such as phosphorylation of protein kinase C, on the growth of certain cells and on the transcription of certain genes (CD36 and collagenase) have been reported.^276^ Plasma level of vitamin E (α-tocopherol) was decreased in patients with NASH.^277^ It was also reported that in patients with NAFLD, vitamin E inhibited TGF β expression in the liver, which reduced steatosis, inflammation, and fibrosis.^278^ In an MCD-induced mouse NASH model, vitamin E supplementation reduced hepatic inflammation and fibrosis by reducing the expression of the proapoptotic BCL2-related X (BAX), TGF-dead, cyclooxygenase-2 (COX-2), and matrix metalloproteinase-2 (MMP-2).^279^ On the other hand, in HFD-fed mice^280^ or humans,^281^ fatty livers produce unrecognized hepatic vitamin E sequestration, which might subsequently drive liver disease, and the sequestered vitamin E might be used to quench oxidants generated within excess fat. These findings indicate that in addition to its antioxidant activity, vitamin E functions in different aspects and mechanisms in NAFL/NASH. Future research should focus more on the detailed molecular mechanism of action of vitamin E to benefit patients with NAFL/NASH.

Currently, vitamin E is recommended to treat NASH patients, associated with reduced serum hepatobiliary enzymes and hepatic steatosis but without improvement of liver fibrosis.^62,282,283^ In a large-sample, randomized, double-blind, controlled, phase 3 clinical trial (NCT00063622), the efficacy of vitamin E with another antidiabetic agent (Pioglitazone, targeting PPARγ) was confirmed in non-diabetic patients with NASH with histological evaluation as the study endpoint. The results showed that the NASH score improvement in the vitamin E group was significantly higher than that in the placebo group (43% vs. 19%), but not significant between the Pioglitazone group (34% vs. 19%) and the placebo group, while combined Pioglitazone and vitamin E improved histological hepatic steatosis and hepatic lobular inflammation without improvement in fibrosis scores.^284^

It has been hypothesized that the responsiveness of NASH patients to vitamin E therapy is affected by Haptoglobin (Hp) genotype. Three randomized controlled trials have shown that diabetic individuals with Hp 2-2, of which patients bearing Hp 2-2 mutation are at increased risk of CVD, had higher efficacy from vitamin E intervention. The percentage of NAFL/NASH Chinese patients with Hp 2-2 allele is much higher than that of western patients (65.71% vs. 36%, respectively), suggesting that Chinese patients may better benefit from vitamin E treatment.^285^ In the long term, vitamin E use is associated with some potential risks such as prostate cancer, stroke, and mortality.^283^ However, due to the differences in the use form of vitamin E and the analysis methods, the conclusions of the current study are uncertain regarding the assessment of risk. Further investigation is required to fully address the efficacy of vitamin E, especially in long-term studies after appropriate analysis.^283–285^

Currently, there are many antioxidants in addition to vitamin E that are being studied for the treatment of NAFLD. For example, the antioxidant carotenoid beta-cryptoxanthin prevented or reversed the progression of steatosis and fibrosis in NASH mice fed a high-cholesterol and high-fat (CL) diet.^286^ Melatonin has also exhibited promising results in patients by controlling the progression of NAFL.^287^ Administration of coenzyme Q_10_ elevated adiponectin levels and decreased MDA levels, suggesting improved lipid peroxidation in patients with metabolic syndrome.^288^ Some other natural dietary antioxidants such as curcumin,^289,290^ green tea, and epigallocatechin gallate^291,292^ also have positive therapeutic effects on NAFL/NASH. Therefore, we believe that even if there are various mechanisms leading to the development of NAFL/NASH, including oxidative stress, antioxidants have the potential to treat NAFL/NASH. Perhaps antioxidants in combination with other drugs may have an unexpected therapeutic outcome.

#### Hepatic cell death and pro-survival

Multiple types of cell death, including apoptosis, necroptosis, pyroptosis, and ferroptosis, as well as autophagy, are associated with the development of NAFL/NASH.^72,293,294^ Among targeting different formats of cell death, inhibition of apoptosis has achieved promising results, here we focus on anti-apoptosis agents (Tables 3 and 4), but other types of cell death will also be discussed below.

**Table 3:** Anti-apoptotic, inflammatory, and fibrogenic agents under clinical trials

Class	Drug classification	Drug name	Registered clinical trails	Outcome	Ref.
Anti-apoptotic agents	ASK1 inhibitor	Selonsertib (GS-4997)	Phase 2 (NCT02466516) Phase 3 (NCT03053050, NCT03053063)	↓Hepatic steatosis ↓Liver fibrosis No antifibrotic effect in patients with bridging fibrosis or compensated cirrhosis due to NASH in phase 3 trials	^321,620^
	Pan-caspase inhibitor	Emricasan (IDN-6556)	Phase 2 (NCT02077374, NCT02230670, NCT02686762)	No effects on liver fibrosis in patients with NASH	^315–317,621^
Anti-inflammatory agents	CCR2/5 antagonist	Cenicriviroc	Phase 2b (NCT02217475) Phase 3 (NCT03028740, terminated)	↓Liver fibrosis ↓Inflammation	^397,398^
	Anti-TNFα drug	Pentoxyfylline (PTX)	Phase 2/3 (NCT00267670, NCT00590161)	↓Hepatic steatosis Effects on NASH are controversial	^408–410^
Antifibrosis	Antioxidant	Vitamin E	Phase 3 (NCT00063622) Phase NA (NCT02962297)	↓Hepatic steatosis ↓Inflammation ↓ALT, AST ↓Liver fibrosis	^284,285,622^
	Angiotensin-II receptor blocker	Losartan	Phase 3 (NCT01051219)	No results posted	
	Galectin-3 (Gal-3) inhibitor	Belapectin (GR-MD-02)	Phase 2 (NCT02462967, NCT02421094) Phase 1 (NCT01899859)	No effects on liver fibrosis in patients with NASH	^434,623^
	LOXL2 antibody	Simtuzumab (SIM, GS-6624)	Phase 2b (NCT01672866, NCT01672879)	Terminated due to lack of efficacy.	^431–433^
	HSP47 inhibitor	BMS-986263	Phase 2 (NCT03420768) (EudraCT Number: 2019-003932-22)	↓Liver fibrosis	^437^
Cell therapy	Stem cell therapy	UC-MSC	Phase 1/2 (NCT01220492)	↓ALB, PTA, CHE, TBIL ↑Survival rate in patients with decompensated liver cirrhosis	^548^
		BM-MSC	Phase 2 (NCT01875081)	Improved histologic fibrosis and liver function in patients with alcoholic cirrhosis	^624^
Genetic approaches	RNAi target HSD17B13	ARO-HSD	Phase 1 (NCT04202354)	↓ALT, AST ↓Hepatic HSD17B13 mRNA and protein	^562^
		ALN-HSD	Phase 1 (NCT04565717)	No results posted	
	ASO against PNPLA3	ION839 (AZD2693)	Phase 1 (NCT04483947, recruiting)	No results posted	
	ASO against DGAT2	IONIS-DGAT2_Rx_	Phase 2 (NCT03334214, NCT04932512, recruiting)	↓Hepatic steatosis	^566^
	GalNAc-conjugated anti-miR-103/107 oligonucleotide	RG-125 (AZD4076)	Phase 1 (NCT02826525, NCT02612662)	No results posted	
Gut microbiota	Gut microbiota targets	Probiotics	Phase NA (NCT00870012)	↓AST ↓Hepatic TG	^530^

**Table 4:** Anti-apoptotic, inflammatory, and fibrogenic agents in the preclinical stage

Class	Targets	Drug name	Experimental models	Outcome	Ref.
Cell death (target on apoptosis)	ASK1 inhibitor	Peptide fragment of CFLAR [CFLAR(S1)]	HFD-fed mouse model Pelleted primate HFD-fed monkey model with MetS predisposal	↓Steatohepatitis ↓Metabolic disorders	^625^
		Overexpression of TNFAIP3 by adeno-associated virus 8 (AAV8)-TNFAIP3 injection	HFD-fed mouse model HFHC diet-fed *ob/ob* mouse model Pelleted primate HFD-fed monkey model with MetS predisposal	↓Lipid accumulation ↓Hepatic steatosis ↓Inflammation ↓Fibrosis	^325^
		Glutathione S-transferase Mu 2 (GSTM2)	HFD-fed *Gstm2-*hepatocyte-specific knockout mouse model	↓Insulin resistance ↓Hepatic steatosis ↓Gene expression related to lipid metabolism	^626^
	Ferroptosis inhibitor	ECH1	MCD diet-fed mouse model	↓Hepatic steatosis, fibrogenesis ↓Inflammation ↓Apoptosis ↓Oxidative stress ↓ALT	^356^
	Cdk4 inhibitor	Flavopiridol or PD-0332991	HFD-fed mouse model	↓Hepatic steatosis	^453^
	Necroptosis inhibitor	TN3-19.12	CD- and HFD-fed mice model	↑RIP1, RIP3, and MLKL ↓ALT and AST ↓Liver injury ↓Hepatocyte necrosis	^336^
		Necrostatin-1 (Nec-1)			
		GSK′872			
	Pyroptosis inhibitor	Cytochalasin B	Primary mouse and human hepatocytes	↓IL-1ß and α-SMA ↓Inflammation and fibrosis	^344^
		Ghrelin	HepG2 cells	↓TNF-α-induced apoptosis and hepatocyte autophagy	^345^
Cell death (target on autophagy)	ATG3 inhibitor	shRNA against ATG3	CDHFD, HFD, or MCD-fed mouse model *TAp63α-*induced mouse steatosis model	↓ALT, AST ↓Steatosis	^326^
	TXNIP agonist	Rapamycin	MCD diet-fed *Txnip*-KO mouse model	↓Steatosis **↓**Inflammation ↓Fibrosis ↓FAO	^330^
Anti-inflammatory agents	Anti-TNF-α drug	Infliximab	MCD diet-fed rat model	↓NASH histopathology score ↓AST, ALT ↓Fibrosis, TGF-β ↓Plasma and tissue MDA ↓Inflammation	^411,412^
		Thalidomide	HFD-fed mouse model	↓Blood glucose level ↓Liver TG content ↓Hepatic inflammatory markers	^413^
	Hepatic macrophage polarization	Annexin A5 (ANXA5)	HFD-fed mouse model	↓Steatosis ↓Inflammation ↓Fibrosis	^627^
	IL-11 antibody	Anti-IL-11 (X203), anti-IL11RA (X209)	High-fat methionine and choline-deficient (HFMCD) diet-fed mouse model MCD diet-fed *db/db* mouse model Western diet supplemented with fructose (WDF)-fed mouse model	↓Hepatic steatosis ↓Fibrosis ↓Inflammation	^402^
	TLR4 antagonist	Sparstolonin B (SsnB)	HFD-fed mouse model	↓Liver fibrosis	^415^
	NLRP3 inhibitor	Sulforaphen (SFN)	HFD-fed mouse model	↓Hepatic steatosis scores ↓Serum ALT and AST ↓Hepatic TC, TG ↓FFAs	^416,417^
	Erk1/2 inhibitor	FR180204	MCD diet-fed mouse model	↓ALT, AST ↓CHO, TG	^628^
	ERK antagonist	Ravoxertinib	MCD diet-fed mouse model	↓ALT, AST ↓CHO, TG ↓Steatosis ↓Inflammatory state	^629^
Antifibrosis	P53 activator	IGF-I	MCD diet-fed *db/db* mouse model Dimethylnitrosamine-treated mouse model	↓Steatosis ↓Inflammation ↓Fibrosis	^630^
	Anti-TGF-β drug	Breviscapine	HFD, HFHC, or MCD diet-fed mouse model	↓Lipid accumulation ↓Inflammatory cell infiltration ↓Liver injury and fibrosis	^448^
		SP-1154	HFD-fed mouse model	↓Inflammation ↑Insulin sensitivity, glucose homeostasis ↓Hepatic steatosis	^441^
		Oxy210	“Western” diet (WD)-fed mouse model	↓Hepatic fibrosis ↓Inflammation ↓Hypercholesterolemia	^442^
		TIPE2	HFD or HFHC diet-fed hepatic Tipe2-transgenic mouse model	↓Hepatic steatosis ↓Insulin resistance ↓Inflammation ↓Fibrosis	^446^
		Tetrodecadazinone	TGF-β1-activated LX-2 cell model	↓Expression of extracellular matrix proteins (fibronectin and collagen I) ↓α-SMA	^443^
	Phosphodiesterase 4 (PDE4) inhibitor	Roflumilast	HFD-fed mouse model	↓Hepatic steatosis ↓Liver weight and body weight ↓Circulating cholesterol (CHO) and LDL-C No effect on circulating HDL-C and TG	^631^
	Medicinal herbs	Yellow loosestrife (*Lysimachia vulgaris* var. davurica)	MCD diet-fed *db/db* mouse model	↓Lipid accumulation ↓Inflammation ↑Antioxidative proteins	^444^
	Suppresses activation of HSCs	Emodin	HSC-T6 cell model	↓Proliferation and activation of HSCs ↓TGFβ1/Smad signaling pathway in activated HSCs	^454,455^
	Overexpression of human HNF4α (hepatocyte nuclear factor 4α)	AAV8-ALB-hHNF4α	High-fat/cholesterol/fructose (HFCF) diet-fed mouse model	↓Steatohepatitis ↓TG ↑FAO ↑VLDL secretion	^632^
	TAZ inhibitor	*GalNAc-siTAZ*	NASH diet-fed mouse model	↓Hepatic inflammation ↓Liver injury ↓Fibrosis	^567^
	Notch inhibitor	GSI	NASH diet-fed mouse model	↓HSC activation and liver fibrosis ↑Goblet cell metaplasia	^568^
		Ncst ASO	NASH diet-fed mouse model	↓Fibrosis (expression of HSC markers and collagen deposition) No effect on serum transaminases and liver inflammation	^568^
Antioxidative stress agents	Antioxidative stress agents	Matrine	HFD or MCD diet-fed mouse model	↓Hepatic inflammation ↓Lipid peroxides ↓ALT and AST ↓Fibrosis	^633,634^
		Polaprezinc	MCD diet-fed mouse model	↓Fibrosis ↓Lipid peroxidation ↓Inflammation No effect on the development of steatosis.	^635^
Genetic approaches	HSD17B13 inhibitor	INI-678	Human cell “3D liver-on-a-chip” model (https://inipharm.com/)	↓Markers of liver fibrosis (*α-SMA, Col-I*)	^562^
	PNPLA3-rs738409 (I148M) variant inhibitor	Minor allele-specific small interfering RNA (siRNA)	NASH diet-fed *PNPLA3*^*I148M*^-expressing mouse model	↓Hepatic TG ↓ALT ↓Histological assessment of inflammation	^636,637^
	PNPLA3 expression downregulator	Momelotinib	Human multilineage 3D spheroid model of NASH homozygous for the PNPLA3 mutant protein	↓*PNPLA3* mRNA ↓Lipid content	^638^
Gut microbiota targets	Gut microbiota targets	Fecal microbiota transplantation (FMT)	HFD-fed mouse model	↓Steatohepatitis ↓NAS score ↓Intrahepatic lipid accumulation ↓Proinflammatory cytokines	^531^
	Adenosine A(2a) receptor agonists	CGS21680	MCD diet-fed rat model	↓ALT ↓Hepatocyte apoptosis ↓Liver inflammation ↓Fibrosis No effect on hepatic steatosis.	^639^
	Ras-association domain family 4 (RASSF4) agonists	Overexpression of RASSF4	*db/db* genetic mouse model	↓Hepatic steatosis ↓Inflammation levels ↓Fibrosis	^640^
	Target sphingomyelin synthase 1 (SMS1)	shRNA-mediated knockdown of *Sms1*	HFHC diet-fed shRNA-mediated knockdown of *Sms1*mouse model	↓Inflammation and fibrosis ↓TGFβ1, α-SMA ↓ALT No effect on hepatic TG	^641^

##### Apoptosis

**Fig. 4:**
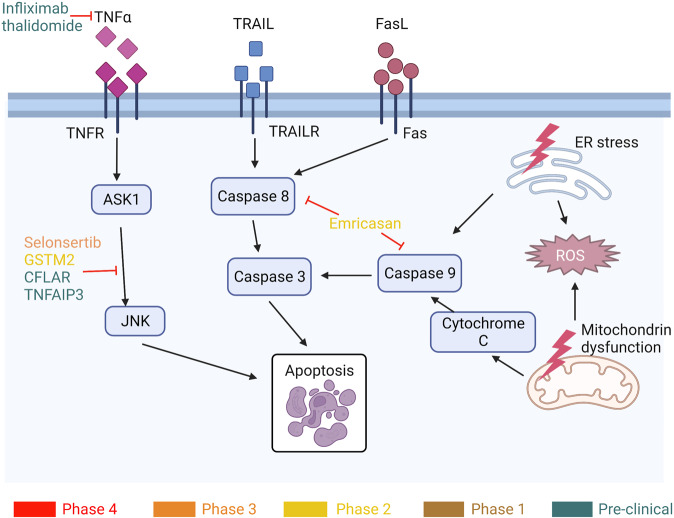
Drugs targeting the apoptosis signaling in NASH. Inflammatory cytokines stimulate hepatocyte apoptosis through different pathways such as TRAIL signaling, Fas signaling and TNFα signaling pathway. TRAIL is a member of the TNF superfamily that can lead to the induction of apoptosis in tumors or infected cells. The Fas receptor induces an apoptotic signal by binding to FasL expressed on the surface of other cells. TNFα is a classical cytokine and its signaling pathway had been well investigated. Antagonists and inhibitors at different trial stages are as indicated. Drugs at different clinical stages are indicated in different colors. Created with BioRender. TRAIL tumor necrosis factor-related apoptosis-inducing ligand, Fas fatty acid synthetase, FADD Fas-associated with death domain protein, FasL Fas ligand, TNFα tumor necrosis factor-alpha, TNFR TNF receptor, TRAF2 TNF receptor-associated factor-2, ASK1 apoptosis signal-regulated kinase 1, JNK c-Jun N-terminal kinase, GSTM2 glutathione s-transferase mu 2, CFLAR caspase 8 and FADD-like apoptosis regulator, TNFAIP3 tumor necrosis factor-alpha-induced protein 3

Hepatocyte apoptosis induced by Caspases,^295,296^ and Bcl-2 family proteins^297,298^ and c-Jun N-terminal kinase (JNK)^299–301^ plays a driving role in the progression of NAFL/NASH. The cytokeratin-18 sheet (CK18) segment generated by Caspase-3 can be used as a marker to predict the severity of NASH.^302,303^ Palmitatic acid stimulation activated tumor necrosis factor-related apoptosis-inducing ligand receptor 2 (TRAIL-R2), leading to caspase-dependent cell death in hepatocytes.^304^ ER stress upregulates proapoptotic proteins, including p53 upregulated modulator of apoptosis (PUMA), Bim, and TRAIL-R2 via CHOP or JNK.^305–307^ PUMA and Bim promote hepatocyte death via the mitochondrial apoptotic pathway. Activated macrophages increase the expression of death receptor ligands, such as Fas ligand (FasL), TNF-related apoptosis-inducing ligand (TRAIL), and proinflammatory cytokines, which further promote hepatocyte apoptosis and inflammatory response.^308–311^ Regulation of miR-34a/SIRT1/p53 signaling by UDCA is involved in hepatocyte apoptosis.^312^ In addition, apoptosis signal-regulated kinase 1 (ASK1), an upstream activator of the JNK and p38 MAPK signaling cascades, can be activated by stress signals, such as ROS, ER stress, and TNFα, and plays a key role in the progression of NASH^313^ (Fig. 4).

Anti-apoptotic agents: Emricasan (IDN-6556) is a pan-caspase inhibitor that reduces inflammation and fibrotic apoptosis. In a murine model of NASH, mice that received Emricasan treatment were protected from liver injury and fibrosis, suggesting that inhibition of hepatocytes apoptosis by Emricasan may be an attractive antifibrotic strategy in NASH.^314^ Furthermore, administration of Emricasan in a phase 2 clinical trials in patients with NASH has shown beneficial results, including the significant reduction of ALT values.^315^ In addition, biomarkers, such as cleaved cytokeratin-18 (cCK18), full-length cytokeratin-18 (flCK18), and caspase-3/7 were significantly decreased in Emricasan-treated subjects (NCT02077374).^315^ Consistently, in another multicenter study of 86 patients with cirrhosis (NCT02230670), serum levels of flCK18 (*p* = 0.02) and caspase-3/7 (*p* < 0.001) were also decreased with 3 months of Emricasan administration compared to the placebo-treated group. Furthermore, at the 3-month timepoint, Emricasan significantly reduced mean MELD (model for ESLD) (*p* = 0.003) and Child-Pugh (*p* = 0.003) scores in subjects whose MELD scores were higher than 15, and significantly reduced international normalized ratio and total bilirubin compared with placebo.^316^ Despite the positive results that caspase inhibition by Emricasan lowered serum ALT in the short term, it may have directed cells to alternative mechanisms of cell death, resulting in more severe liver fibrosis and hepatocyte ballooning (NCT02686762).^317^

Selonsertib (GS-4997), an ASK1 inhibitor, prevents hepatocyte apoptosis and can reverse fibrosis and reduce liver inflammation in different preclinical models.^318,319^ In a 3D in vitro microtissue model, administration of Selonsertib decreased the measurements of specific disease parameters, such as the secretion of the profibrotic factor (procollagen type I), proinflammatory cytokines (TNF-α and IL-6), and chemokines (MCP-1, MIP-1α, IL-8, IP-10), in accordance with clinical observations.^320^ In a short-term phase 2 trial, the administration of Selonsertib improved NASH and fibrosis (at least a one-stage improvement) in some patients (NCT02466516).^247,248^

Two randomized, double-blind, placebo-controlled, phase 3 trials of Selonsertib in patients with NASH and bridging fibrosis (F3, STELLAR-3) or compensated cirrhosis (F4, STELLAR-4) were conducted (NCT03053050 and NCT03053063);^321^ however, neither trial met the primary efficacy endpoint. In STELLAR-3 (NCT03053050), fibrosis improvement without worsening of NASH was observed in 10% (18 mg, *p* = 0.49 vs. placebo), 12% (6 mg, *p* = 0.93 vs. placebo), and 13% (placebo) of patients. In STELLAR-4 (NCT03053063), the primary endpoint was achieved in 14% (18 mg, *p* = 0.56), 13% (6 mg, *p* = 0.93), and 13% (placebo) of patients.^321^ Although Selonsertib led to dose-dependent reductions in hepatic levels of phosphorylated p38, an indicator of its pharmacodynamic activity, it does not have a significant effect on liver biochemistry, non-invasive tests of fibrosis, progression to cirrhosis, or adjudicated clinical events, probably due to the advanced fibrosis onset in these patients.^321^

A small peptide segment in caspase 8 and FADD-like apoptosis regulator (CFLAR) that effectively attenuates the progression of steatohepatitis and metabolic disorders in both mice and monkeys. The dimerization and subsequent autophosphorylation of ASK1 are essential for its activation.^322,323^ CFLAR directly targets ASK1 and interrupts its N-terminus-mediated dimerization, thereby blocking signaling involving ASK1 and JNK1.^324^ Tumor necrosis factor-alpha-induced protein 3 (TNFAIP3) is a pivotal endogenous suppressor of ASK1 hyperactivation in the pathogenesis of NASH. Hepatocyte-specific ablation of TNFAIP3 exacerbated NAFLD- and NASH-related phenotypes in mice, including glucose metabolism disorders, lipid accumulation, and enhanced inflammation, in an ASK1-dependent manner.^325^ In addition, hepatic knockdown of autophagy-related gene 3 (ATG3) by i.v. injection of lentivirus encoding shRNA against ATG3 ameliorates the development of NAFL/NASH by stimulating mitochondrial function.^326^ Accordingly, ATG3 was identified as a new target of NAFL/NASH downstream of p63, and activation of p63 induced hepatic steatosis in diet-induced obese mice.^326,327^ In addition, it was reported that the upregulation of the p53 protein in hepatocytes might be a therapeutic strategy in the treatment of NAFL/NASH, for example, pharmacological stimulation of p53 with low-dose Doxorubicin ameliorates diet-induced nonalcoholic steatosis and steatohepatitis.^328,329^ Similarly, IGF-I induces senescence of HSCs, inactivates these cells, and limits fibrosis in a p53-dependent manner.

Rapamycin (a thioredoxin interacting protein (TXNIP) agonist) treatment also attenuated MCD-induced steatosis, inflammation, and fibrosis with increased TFEB nuclear translocation and restored FAO in TXNIP-KO mice by inhibiting MTORC1 to promote autophagy.^330^ Despite the positive preclinical results, more clinical studies should be conducted to verify the efficacy of apoptosis inhibitors in the treatment of patients with NAFL/NASH.

##### Necroptosis

Necrotizing apoptosis or necroptosis has been identified as a key pathogenic mechanism in NAFL/NASH. For example, upon TNFα stimulation, Receptor-interacting serine/threonine protein kinase-3 (RIP3) RIP3 phosphorylates mixed lineage kinase domain-like (MLKL) and induces cell necroptosis. In general, RIP3, RIP1, and MLKL together form necrosome, and hepatic RIP3 and MLKL phosphorylation and TNFα expression are increased in NAFL/NASH.^331^ Inhibition of MLKL, RIP3, or RIP1 all improved the NASH characteristics of HFD-fed mouse models.^332,333^ In addition, MLKL-dependent (but RIP3-independent) noncanonical signaling contributes to western diet-induced liver injury by inhibiting autophagy and inducing necrosis.^332,334,335^ TN3-19.12, a neutralizing monoclonal antibody against TNFα, could inhibit necroptosis that was activated in NAFLD, with the upregulation of RIP1, RIP3, and MLKL in both CD- and HFD-fed mice. In addition, necroptosis inhibitors necrostatin-1 (Nec-1) and GSK-872 could also inhibit necroptosis in both CD- and HFD-fed mice significantly.^336^

##### Pyroptosis

Pyroptosis is characterized by the formation of inflammasome, activation of Caspases and Gasdermin, and the release of proinflammatory cytokines, including IL-1β and IL-18. The canonical pyroptosis pathway recruits and activates caspase-1 upon recognition of PAMPs and DAMPs.^337^ In noncanonical pyroptosis, caspase-1/11 in mice and caspase-4/5 in human is stimulated directly by LPS in a TLR4-independent manner.^338,339^ These activated Caspases cleave and activate Gasdermin D (GSDMD) to promote the secretion of proinflammatory cytokines (IL-1β, TNF-α, and MCP-1/CCL2) and activation of NF-ĸB signaling pathway.^340^ In addition, expression of SREBP1 is reduced in MCD-fed GSDMD-deficient mice, upregulating lipolytic genes and resulting in reduced adipogenesis, subsequently, attenuated MCD-fed induced NASH.^341^ Increased activity of caspase-1, GSDMD, and inflammasome components was observed in mice with MCD-induced NASH.^342^ However, there was no activation of inflammasome in mice with simple steatosis, suggesting its role in more severe disease or the progression to NASH. The nucleotide-binding oligomerization domain-like receptor family pyrin domain-containing 3 (NLRP3) inflammasome blockade normalized hepatic caspase-1 and IL-1β expression and reduced liver fibrosis in MCD diet-fed mice.^343^ Cytochalasin B, an endocytic inhibitor, eliminated the increased secretion of inflammatory factor IL-1β and the expression of α-smooth muscle actin (α-SMA) in HSCs that internalized NLRP3 inflammasome particles.^344^ Ghrelin is a gut hormone with 28-amino acid peptide. Two ghrelin isoforms (acylated ghrelin and desacyl ghrelin) were protective against high-mobility group box 1 (HMGB1) induced pyroptosis in HepG2 cells.^345^

##### Ferroptosis

It is reported that patients with NAFL/NASH frequently suffer from hypoferritinemia and are more likely to develop advanced fibrosis, or even have a higher mortality rate compared with NAFL/NASH patients with a normal level of ferritin.^346,347^ Growing evidence suggest that iron-dependent ferroptosis plays an essential role in the development of multiple liver diseases, including NAFL/NASH.^348–350^ Ferroptosis refers to a type of iron-dependent programmed cell death that is characterized by the accumulation of lipid peroxide and ROS derived from iron metabolism, which is genetically and biochemically distinct from other forms of regulated cell death.^351,352^ It was reported that scavenging the associated lipid peroxidation in hepatocytes with ferroptosis inhibitors could reduce lipid accumulation and alleviates MCD-induced mouse NASH model and liver fibrosis.^350^ Glutathione peroxidase 4 (GPX4) is an antioxidant defense enzyme, whose function is to repair oxidative lipids damage and is a leading inhibitor of ferroptosis.^353^ Activation of GPX4 protected mice from HFD-induced obesity and improved hepatic steatosis in mice.^354^ Mechanistically, activation of the Keap1/Nrf2 pathway promotes downstream gene expression of heme oxygenase-1 (HO-1), glutathione (GSH), and its peroxidase 4 (GPX4), which eliminate ROS accumulation, inhibit gluconeogenesis and adipogenesis, thereby reducing NAFL/NASH.^349,355^ Furthermore, activation of GPX4 by Enoyl coenzyme A hydratase 1 (ECH1) suppressed hepatic ferroptosis and significantly alleviated hepatic steatosis, inflammation, fibrogenesis, apoptosis, and oxidative stress in livers of mice fed with MCD diet.^356^ There are other pathways that alter the susceptibility to ferroptosis, including amino acid and iron metabolism, ferritin autophagy, cell adhesion, p53, and phospholipid biosynthesis.^357–360^ In addition, the RNA-binding protein ELAVL1/HuR, ZFP36/TTP, and the bromodomain-containing protein 7 (BRD7) have also been shown to impact liver fibrosis by regulating ferroptosis in HSCs.^361–363^

#### Hepatic inflammation in NASH/NAFL/NASH and anti-inflammatory therapy

Cell death can be both a consequence and a cause of inflammation.^364^ While apoptosis induces minimum inflammatory response during the progression of NAFL/NASH, lytic forms of cell death, such as necroptosis and pyroptosis, can trigger the inflammation via DAMPs.^293^ On the other hand, the extensive release of inflammatory mediators, such as TNFα and TGFβ, will cause more cell death, which collectively promotes the pathogenesis of NAFL/NASH.

##### Regulation of inflammatory response in NAFL/NASH

**Fig. 5:**
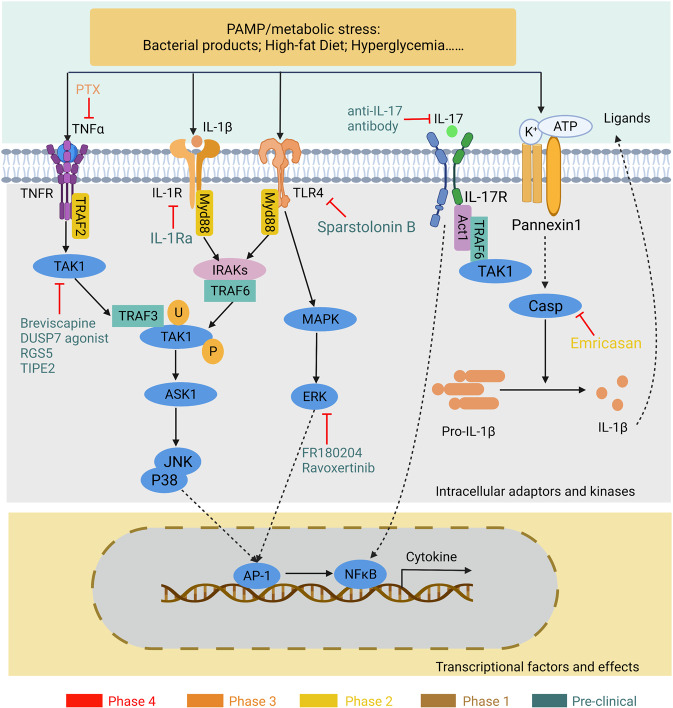
Drugs regulating the inflammatory response in NAFL/NASH. In NASH, extracellular PAMPs or metabolic stress activates proinflammatory signaling pathways through multiple receptors. Drugs regulate inflammation in NAFL/NASH by targeting different inflammatory pathways, such as TNF-α, TLR-IL-1R, IL-17 signaling, and caspase signaling. Drugs at different clinical stages are indicated in different colors. Created with BioRender. PAMP pathogen-associated molecular patterns, TLR Toll-like receptor, IL-R interleukin-receptor, Myd88 myeloid differentiation factor 88, IRAK interleukin-1 receptor-associated kinase 1, TRAF TNF receptor-associated factor, ASK apoptosis signal-regulating kinase, TAK transforming growth factor beta-activated kinase, JNK c-Jun N-terminal kinase, MAPK mitogen-activated protein kinase, ERK extracellular signal-regulated protein kinases, Casp caspase, AP-1 activating protein-1, NFκB nuclear factor kappa B, PTX pentoxifylline, DUSP7 dual specific phosphatase, RGS5 hepatic regulator of G protein signaling 5, TIPE2 tumor necrosis factor-alpha–induced protein 8-like 2. Drugs at different clinical stages are indicated in different colors

Inflammatory response is an essential contributor to the development and progression of NAFL/NASH. Immune cells and proinflammatory cytokines are implicated to play significant roles in NASH pathogenesis (Figs. 2 and 5).

KCs, liver-resident macrophages, are the first line of sensors in the liver and are critical in the development of NAFL/NASH.^365–367^ Both the release of gut flora-derived bacterial products (e.g., LPS, bacterial DNA)^25^ due to increased intestinal permeability and exposure to endogenous substances (e.g., HMGB1, FFAs)^368,369^ from damaged cells activate hepatic TLRs (e.g., TLR4, TLR2, and TLR9) and NLRs on KCs. The downstream signaling events increase nuclear translocation of NF-κB, further promoting the production of various proinflammatory and profibrogenic cytokines, such as TNFα, IL-1β, CCL5, and TGFβ, which subsequently induce hepatocyte lipid accumulation and apoptosis and HSC activation.^365,370^ Fatty acids can promote the release of mtDNA or cholesterol crystals leading to activation of NLRP3 inflammasome in KCs that secrete the proinflammatory cytokine IL-1β.^343,371^ Activated KCs also promote the recruitment of monocyte-derived macrophages (MoMFs) to the liver in a CCR2-dependent manner by secreting CCL2.^372–374^ The recruited inflammatory MoMFs further amplify the inflammatory response. The number of macrophages is positively correlated with NAFL/NASH disease severity, and more macrophages are found even at the early stages of human NASH. Moreover, during NASH, hepatocytes upregulate the main neutrophil recruiting chemokines (CXCL1 and IL-8),^375^ and neutrophil infiltration around the lipotoxic hepatocytes is considered a hallmark of NASH.^376,377^ Neutrophil abundance correlates with the degree of steatosis and fibrosis. Inhibition of Neutrophils derived granule proteins and neutrophil extracellular traps could reduce macrophage infiltration and inflammatory cytokine secretion, decreasing the progression of NASH to HCC in NASH mice induced by neonatal streptozotocin and HFD.^378^ Myeloperoxidase (MPO), an important neutrophil enzyme, contributes to HSC activation and is proapoptotic and profibrotic in NASH.^379^ Increased expression and activity of neutrophil elastase (NE) in NASH lead to hepatocyte IR and are partially associated with the regulation of hepatic ceramide metabolism.^380–382^ Neutrophil-derived lipocalin 2 (LCN2) exacerbates steatohepatitis by inducing CXCR2 expression to promote crosstalk between neutrophils and hepatic macrophages.^383^ In addition, adaptive immunity is also involved in the development of NAFL/NASH.^384^ Interestingly, Th17 development is dependent on ACC1-mediated de novo fatty acid synthesis and potentially glycolytic lipogenic metabolic pathways, demonstrating crosstalk between pathways like lipogenesis and inflammation.^385^ Increased Th17 cells and expression of IL-17A are markers of progression from NAFL to NASH.^386,387^ CD4^+^ T cells may also regulate the inflammatory response of macrophages through IFNγ, affecting liver fibrosis.^388^ In NASH, pathogenic CD8^+^ T cells accumulate in the liver.^389^ These accumulation and activation of T cells and myeloid cells are largely associated with a type I interferon (IFN-I) response in the liver, leading to increased production of the proinflammatory cytokines IFNγ and TNFα.^390^ At the early stage of NAFL, B-cell responses precede T cells and are accompanied by upregulation of hepatic expression of B-cell-activating factor (BAFF).^391^ In addition, activated B cells produce pro- or anti-inflammatory cytokines, immunoglobulins, activate T cells, and may also exacerbate tissue damage and liver fibrosis.^392–394^ In short, there are complex immune mechanisms during NAFL/NASH; however, the current understanding of the inflammatory response driving NASH is fragmented. Therefore, more intensive research and conclusive evidence are needed to understand the role of immune cells during disease progression.

##### Anti-inflammatory therapy

Treatment with C-C chemokine receptors (CCR) antagonists to avoid the abnormal infiltration of inflammatory leukocytes can also be used as a therapy for NASH.^395^ In a mouse model of diet-induced NASH and a rat model of thioacetamide (TAA)-induced liver fibrosis, CCR2/5 antagonists (Cenicriviroc, CVC) effectively reduced fibrosis as measured by reducing protein expression of collagen I and α-SMA, and collagen deposition in the region around the liver lobules by targeting hepatic-pathogenic monocytes/macrophages and HSCs.^396^ CVC further underwent a phase 2 clinical evaluation (NCT02217475) and the results indicated that CVC improved fibrosis and had greater efficacy in advanced fibrosis.^397,398^ CVC at year 2 achieved ≥1-stage fibrosis improvement (24% of patients in the CVC group and 17% in the placebo group) and no worsening of NASH (*p* = 0.37).^396,397^ In addition, 60% of patients on CVC who achieved fibrosis response at year 1 maintained benefit at year 2, including 86% on CVC who had stage 3 fibrosis at baseline.^397^ Moreover, CVC treatment has antifibrotic and anti-inflammatory functions by reducing multiple systemic inflammation biomarkers, including C-reactive protein, IL-6, IL-1β, and fibrinogen.^397^

Expanding evidence suggested that targeting proinflammatory cytokines exhibited protective effects on NAFL/NASH in preclinical trials, including IL-27, IL-17, IL-11, IL-1, and TNFα, ensemble a significant portion of anti-inflammatory strategy to treat NAFL/NASH. The IL-27 receptor (IL-27Ra, or WSX-1) expresses on adipocytes and plays an important role in promoting metabolic diseases. In addition, IL-27 expressed on T cells also provides new targets for the treatment of obesity-related metabolic syndrome, such as NAFL/NASH.^399^ Administration of recombinant mouse IL-27 ameliorated metabolic morbidities in obese mice by activating p38 MAPK-PGC-1α signaling axis and stimulating the production of uncoupling protein-1 (UCP1).^400^ IL-17 stimulates KCs to express the major fibrogenic cytokine TGF-β1, directly induced production of collagen type I in HSCs by activating the signal transducer and activator of transcription 3 (Stat3) signaling pathway, and may serve as an attractive target for antifibrotic therapy.^401^ Upon anti-IL-17 antibody treatment, the development of hepatic fibrosis was alleviated in NASH mice.^401^ Anti-IL-11 (X203) and anti-IL-11RA (X209) antibodies reduced fibrosis, steatosis, hepatocyte apoptosis, and inflammation in mice with diet-induced liver steatosis and fibrosis by IL-11-induced ERK-mediated signaling pathway.^402^ Moreover, it was reported that IL-1 was a key proinflammatory factor, including IL-1α and IL-1β, which promoted IR by impairing adipocyte function and promoting inflammation.^403^ Hepatic macrophage KCs upregulate IL-1α expression and recruit neutrophils and monocytes to sites of inflammation, exacerbating inflammation and injury. Thus, IL-1 became a key target for the treatment of obesity-related inflammatory diseases, such as NASH. As a major antagonist, IL-1Ra inhibits the IL-1R signaling cascade by preventing the binding of IL-1α/IL-1β, and subsequently attenuates the inflammatory response.^403,404^ Various natural and synthetic drugs have been found to exert anti-NASH effects by targeting the IL-1 signaling pathway. Natural flavones with anti-inflammatory effects showed the protective properties of NASH in obese mouse models by targeting IL-1 and IL-18.^405,406^ A recent clinical trial has demonstrated that diacerein (1,8-diacetoxy-9,10-dioxo-dihydroanthracene-3-carboxylic acid), an immune-modulator anti-inflammatory drug, mainly acting on IL-1 pathways, remarkably decreased liver fibrosis in diabetic patients with NAFLD.^407^ Pentoxyfylline (PTX), an anti-TNFα drug, failed to reduce transaminases and did not positively affect any of the metabolic markers postulated to contribute to NASH, but improved histological features of NASH patients.^408–410^ Although Infliximab, a TNFα inhibitor, did not prevent the development of NASH, it was able to slightly reverse the NASH histopathology score and was effective on necrosis, inflammation, and fibrosis in the NASH rat induced by the MCD diet.^411,412^ In addition, an immunosuppressant drug (Thalidomide) that targets the expression of TNFα may also contribute to reductions in the inflammatory markers that were associated with obesity in Swiss mice fed with HFD.^413^

In addition, other proinflammatory pathways served as potent mediators of inflammation and could be potential targets for therapy. ERK antagonists such as FR180204 and Ravoxertinib could significantly reduce ALT, AST, CHO, and TG values in an MCD mice model, and observably improve fatty degeneration, inflammatory state, and liver injury.^414^ Sparstolonin B (SsnB), a polyphenol, attenuates liver fibrosis in rat primary HSCs and human transformed HSCs (LX-2) by antagonizing TLR4-mediate TGFβ signaling pathway.^415^ Sulforaphane (SFN) could suppress NLRP3 inflammasome by regulating AMPK-autophagy axis.^416,417^ SFN reduced ALT and AST levels in serum, hepatic steatosis scores, and other hepatic indicators such as cholesterol, TG, and FFAs levels in mice fed with an HFD.^416^ Purinergic receptor P2X7 (P2RX7) is a major driver of NLRP3 inflammasome activation and IL-1β processing. SGM-1019 (EVT-401), a P2RX7 inhibitor, improved histological characteristics of NASH and protected from liver inflammation and fibrosis in a CCl_4_-induced nonhuman primate model of liver fibrosis.^418^

#### Fibrosis

**Fig. 6:**
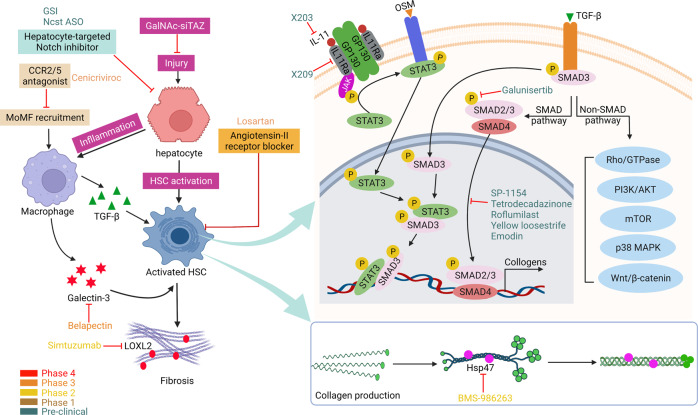
Drugs targeting the fibrosis process in NASH. Chronic hepatocyte injury induces the activation of hepatic stellate cells (HSCs) and the recruits of immune cells, which result in the deposition and cross-linking of collagens in the extracellular matrix and eventually progress to fibrosis. TGFβ/SMAD signaling, a key pathway in the development of liver fibrosis and inflammation, activates Smad pathway and no-Smad pathway. Activation of TGF-β signaling with OSM exposure drives a cooperative STAT3/SMAD3 gene transcriptional program. OSMR/JAK-mediated STAT3 signaling promotes liver fibrosis and HSCs activation by phosphorylation of SMAD3, resulting in transcriptional activation of select STAT3/SMAD3 targets. Antagonists are indicated with a red inhibitor. Drugs at different clinical stages are as indicated. Drugs at different clinical stages are indicated in different colors. Created with BioRender. Ncst ASO nicastrin antisense oligonucleotide, CCR2/5 C-C chemokine receptor 2/5, MoMF monocyte-derived macrophages, TGF-β transforming growth factor-β, LOXL2 lysyl oxidase-like 2, HSC hepatic stellate cell, OSM oncostatin M, IL-11 interleukin-11, IL-11R IL-11 receptor, GP130 glycoprotein 130, JNK c-Jun N-terminal kinase, STAT3 signal transducer and activator of transcription 3, hsp47 Heat shock protein-47

Stressed or damaged hepatocytes and activated macrophages (KCs) lead to the activation of HSCs to myofibroblasts that drive hepatic fibrogenesis. The main feature of liver fibrosis is the deposition of type I collagen in the ECM, which disrupts the normal physiology of the liver and leads to liver dysfunction. HSCs are the main source of ECM-producing myofibroblasts in models of fatty liver disease (Figs. 2 and 6).^419^ TGF-β is one of the most potent profibrotic cytokines in the liver fibrosis process, which mediates the transformation of HSCs to hepatic fibroblasts.^420^ In the TGF-β signaling pathway, Smads, activated type I receptor phosphoplasmic proteins, are the direct acting substrates of TGF-β.^421^ TGF-β binds to the type II receptor TGFβR2 (TβR2) and phosphorylates the N-terminal glycine-serine rich GS domain of type I receptor TGFβR1 (TβR1), whose phosphorylation enables activation of TβR1 and the binding of Smad. Activated TβR1 then phosphorylates and acetylates Smad2/3, which is in complex with Smad4, translocates to the nucleus, and promotes profibrotic gene expression.^422^ In addition to Smad-dependent pathways, TGF-β can also activate liver fibrosis via noncanonical (non-Smad) pathways such as MAPK, mTOR, PI3K/Akt, IKK, Wnt/β-catenin, and Rho GTPase pathways.^423–425^ Inhibition of TGFβ reduces NASH-induced fibrosis and is more effective when combined with IL-13 inhibition.^426^ The oxidative hepatic environment in obesity inactivates the STAT1/3 phosphatase TCPTP (T-cell protein tyrosine phosphatase) and increases STAT1 and STAT3 signaling. Of interest, STAT1 and STAT3 have segregated roles in driving NASH, fibrosis, and HCC. Activation of STAT1 promotes T-cell recruitment, NASH, and fibrosis, but not HCC, whereas inhibition of STAT3 signaling prevented HCC without affecting NASH and fibrosis,^427^ suggesting the possibility of separative treatment of NASH and HCC. IL-17 also directly activates HSCs^428^ and promotes collagen production via the STAT3 pathway.^401^ In addition, platelet-derived growth factor (PDGF) is also a fibrosis-promoting cytokine. HSCs express high levels of PDGF receptors, and their activation effectively stimulates HSC proliferation and migration.^420,429^

##### Antifibrosis agents

The control or reversal of liver fibrosis is important for NASH treatment. Drugs designed to directly antifibrosis (e.g., galectin-3 inhibitor GR-MD-02) or to increase ECM turnover rates (e.g., Simtuzumab) are currently in clinical trials. Lysine oxidase-like 2 (LOXL2) is a matrix formation enzyme that is highly expressed in fibrotic regions of the liver that promotes liver collagen and elastin cross-linking.^430^ However, Simtuzumab (a LOXL2 inhibitor) did not significantly decrease fibrosis stage or the progression to cirrhosis in patients. Two phase 2b clinical trials of Simtuzumab were all terminated after 96 weeks due to the lack of efficacy (NCT01672866 and NCT01672879).^431–433^ Galectin-3 (Gal-3) is a β-galactoside binding protein expressed in immune cells that recognizes and binds to galactose residues and is associated with chronic inflammation and fibrogenesis. GR-MD-02 (Belapectin), an inhibitor of Gal-3, reduced hepatic fibrosis and portal hypertension in rats. In a phase 2 clinical trial in patients of NASH with cirrhosis and portal hypertension (NCT02462967), Belapectin was not associated with significant fibrosis reduction.^434^ The current data indicated that Belapectin might be beneficial in the early stages of cirrhosis, the ongoing phase II b/III study (NCT04365868, NCT02421094) in NASH with cirrhosis or advanced fibrosis patients will further investigate the potential benefits of Belapectin.^435^ Heat shock protein-47 (Hsp47) is a collagen-specific molecular chaperone, which plays an essential role in collagen synthesis and deposition to promote fibrosis.^436^ BMS-986263, a lipid nanoparticle delivering small interfering RNA (siRNA) of HSP47 could promote HSC apoptosis, thereby treating advanced fibrosis.^437^ Several clinical trials of BMS-986263 for advanced fibrosis with different causes have been carried out (NCT02227459, NCT03420768). In addition, Losartan, an angiotensin-II type I receptor antagonist, improved hepatic fibrosis by inhibiting the activation of HSCs in mice models and is now evaluated in a phase 3 trial (NCT01051219) for NASH patients with steatohepatitis and fibrosis (Kleiner fibrosis classification F1-F3).^438^

As a significant player in NASH fibrosis, TGF-β has been one of the actively studied drug targets for antifibrosis. Multiple anti-TGF-β signaling compounds showed promising efficacy in the preclinical studies. Galunisertib (LY2157299), a TGF-β receptor type I kinase inhibitor, showed similar antifibrotic potency in human and rat livers by inhibiting the phosphorylation of SMAD2.^439,440^ SP-1154, a synthetic TGF-β inhibitor, significantly improved insulin sensitivity with glucose homeostasis and reduced hepatic steatosis by inhibiting TGF-β/Smad3 signaling pathway.^441^ Oxy210, a novel inhibitor of hedgehog and TGF-β signaling, effectively ameliorated hypercholesterolemia, hepatic fibrosis, and inflammation in mice fed with a western diet.^442^ Tetrodecadazinone (a novel tetrodecamycin-pyridazinone hybrid) reduced the ECM proteins (fibronectin and collagen I) and α-smooth muscle actin (α-SMA) levels by regulating TGF-β1/Smad2/3 signaling pathway.^443^ Yellow loosestrife (*Lysimachia vulgaris var. davurica*) extracted strongly prevented liver fibrosis by blocking TGFβ/Smad signaling in mice.^444^ The noncanonical TGF-β-activated kinase 1 (TAK1) was shown to be a major upstream signaling molecule in TGF-β1-induced type I collagen and fibronectin expression.^445^ TNFα-induced protein 8-like 2 (TIPE2) suppresses NAFL/NASH advancement by blocking TAK1-JNK/p38 pathway and is a promising target molecule for NAFL/NASH therapy.^446^ Dual specific phosphatase 7 (DUSP7) deletion considerably promoted the activation of TAK1 and might function as a protective factor against NAFL/NASH development through alleviating dyslipidemia, inflammation, and oxidative stress by directly interacting with TAK1 in hepatocytes, which was involved in the suppression of fibrosis.^447^ Breviscapine, a crude extract of several flavonoids of *Erigeron breviscapus (Vant.) Hand.-Mazz*., prevented metabolic stress-induced NASH progression and significantly reduced lipid accumulation, inflammatory cell infiltration, liver injury, and fibrosis in mice through direct inhibition of TAK1 signaling.^448^

Alternatively, reducing the number of activated HSCs by promoting their apoptosis or increasing their clearance is another approach to reduce hepatic fibrosis. The apoptosis inducers, such as fraxetin and 4-hydroxy-2(3H)-benzoxazolone have been shown to induce HSC apoptosis in many animal experiments.^363,364^ Activated HSCs could be killed by the immune surveillance ability of natural killer (NK) cells, suggesting therapeutic activation of NK cells could be an approach to scavenge activated HSCs.^449,450^ However, as hyperactivated NK cells could also enhance inflammation and lead to the progression of fibrosis, specifically targeting the key pathogenic HSCs within the liver is important for this strategy. IFNγ, a potent anti-fibrogenic cytokine produced by NK cells, was conjugated to a cyclic peptide recognizing the platelet-derived growth factor beta receptor (PDGFβR) that was found strongly upregulated on activated HSCs. The IFNγ conjugates attenuated local HSC activation in an acute liver injury model and inhibited fibrogenesis in the liver fibrosis model, with no observed IFNγ-related side effects.^451^ Cyclin-dependent kinase 4 (CDK4) is an important regulator to phosphorylate C/EBPα at Ser193 and increase the formation of C/EBPα-p300 complexes, which regulate the development of hepatic steatosis.^452,453^ Inhibition of CDK4 by Flavopiridol or PD-0332991 significantly attenuated HFD-induced hepatic steatosis and fibrosis in mice, indicating that targeting CDK4 might be an appropriate strategy for resolving liver fibrosis.^453^ In addition, Emodin was reported to suppress the activation of HSCs and ECM-related gene expression in HSCs (HSC-T6) via blocking SMAD4 and p38 MAPK signaling pathways.^454,455^

## Novel signaling pathways and pharmacological targets

While a vast body of studies explained the genetic, biochemical, immunological, and molecular mechanisms responsible for the onset and progression of NAFL/NASH, emerging new insights have been described regarding the signaling pathways that participate in NAFL/NASH pathogenesis, which may shed light on individualized approaches for future management of NAFL/NASH.

### Extracellular vesicles

EVs have shown an increasingly important role as a mode of intercellular communication in regulating tissue and intercellular metabolic signaling.^456^ Circulating EVs have increased in human NASH samples as well as mouse models of NASH.^457,458^ The critical role of EVs in the progression of NAFL/NASH has also been described in more detail in other reviews.^459,460^ Under both normal and pathological conditions, EVs act as communication mediators between the liver and other organs, carrying various bioactive molecules, including lipids, proteins, DNA, coding, and non-coding RNAs.^461^ The increased level of hepatocyte-derived EVs correlates with the severity of NASH;^462^ therefore, the number of circulating EVs and their protein and miRNA composition might potentially be a powerful tool for NAFL/NASH diagnosis and treatment.^462,463^ During lipid overload, miRNA-containing EVs derived from hepatocytes, including let-7e-5p and miR-210-3p, target adipocytes to promote lipid accumulation.^464^ miRNAs in EVs released from lipotoxic hepatocytes (miR-128-3p is most effective) suppress PPARγ expression in HSC, leading to significantly upregulated expression of fibrotic genes, including *Col1a1* and *Atca1.*^465^ Interestingly, neutrophil-derived miR-223-rich EVs target hepatocytes to limit the progression of NASH.^466^ Bone marrow-specific IL-6 signaling also inhibits liver fibrosis through exosomal miR-223 transfer to hepatocytes.^467^ Notably, human liver stem cell (HLSC)-derived EVs have therapeutic actions to NASH,^468^ and serum EVs from normal healthy individuals can decrease the fibrosis-associated molecule expression in activated HSC.^469^ In the liver of patients with NASH, hepatocytes release ceramide-rich inflammatory EVs to recruit MoMFs after activation of IRE1α in ER leading to inflammation and injury.^470^ miR-192-5p-rich exosomes released from hepatocytes induce activation of proinflammatory macrophages by regulating Rictor/Akt/FoxO1 signaling.^471^ In addition, hepatocyte-derived EVs activate NLRP3, inhibit KLF4 and activate NF-κB to increase vascular permeability and promote vascular endothelial inflammation.^472,473^ This suggests a novel mechanism of the association of NAFL/NASH with CVD. Overall, EVs in NAFL/NASH act as a signaling mediator, leading to lipid accumulation, macrophage, and HSC activation, promoting inflammation and liver fibrosis progression, EVs may serve as new targets for the treatment of NAFL/NASH.

### Gut–liver axis

**Fig. 7:**
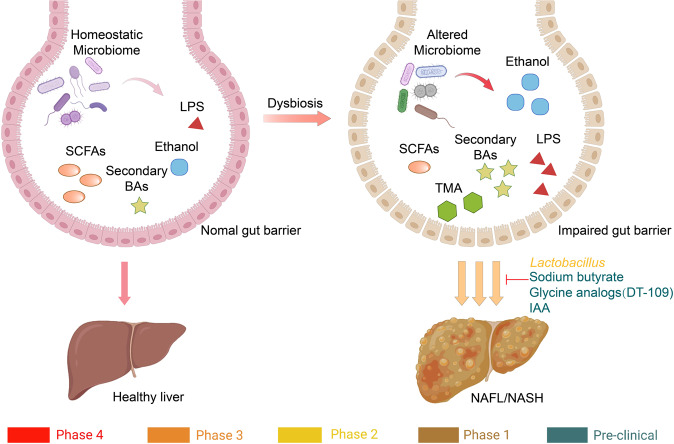
Gut–liver axis in NAFL/NASH. Unhealthy diet, such as high fat and high sugar, induces changes in the intestinal microbiome. This in turn affects alterations in metabolites, such as a decrease in beneficial SFAs and an increase in LPS, ethanol, TMA, etc. The impaired gut barrier allows increased and easier translocation of these dangerous substances to the liver, accelerating the progression of NAFL/NASH. BAs bile acids, SCFAs short-chain fatty acids, LPS lipopolysaccharide, TMA trimethylamine, IAA indole-3-acetic acid

The gut–liver axis is bidirectional communication between the gut and the liver.^474^ Liver influences the composition and function of the gut microbiota and regulates the intestinal barrier through bile duct excretion of BAs and inflammatory mediators. On the other hand, however, the gut microbiota and its metabolites act on the liver through the portal vein to regulate BAs synthesis and hepatic glucose and lipid metabolism.^475^ Disturbances in the gut–liver axis have a key role in the pathogenesis of NAFL/NASH, including alteration in the gut microbiota,^476,477^ disruption of the gut barrier,^478^ and the liver inflammatory response^479^ (Fig. 7).

Changes in the levels and composition of BAs modulate the composition of the gut microbiota.^480^ External administration of BAs simplified the composition of the microbiota and resulted in a significant decrease in serum levels of lipocalin in rats.^187^ Genetic and pharmacological interference with BA synthesis and excretion can alter the bacterial composition of the intestine. For example, oral administration of glycine-β-muricholic acid (Gly-MCA) alters the gut microbial community structure, decreases the ratio of *Firmicutes* to *Bacteroidetes*, reduces the substrate for hepatic DNL, and improves obesity-related metabolic dysfunction.^481^ In addition, inhibition of intestinal bacterial bile salt hydrolases activity also improved glucose and adipogenesis in the organism.^482^

Gut microbiota regulates the synthesis of BAs through FXR.^483^ It was reported that BA diversity is lower in sterile and antibiotic-treated rats, whereas the proportion of BAs bound to taurine, a conjugated form that can increase the solubility and lipid-emulsification properties of BAs, is elevated in peripheral tissues.^484^ During the use of acarbose in the treatment of diabetes, the microbiome in the gut of patients changed, typically the ratio of bound BA to unbound BA in plasma increased.^485^ The use of taurine deoxycholic acid, a BA derivative, improved ER stress and increased insulin sensitivity in the liver and muscle.^486^ Germ-free mice fed with an HFD of cholesterol-rich lard have increased energy expenditure and fecal fat excretion, prioritized carbohydrate oxidation, and reduced fat accumulation.^487^

Although *Firmicutes* and *Bacteroidetes* remain the dominant phylum, the proportions of *Bacteroidetes* to *Firmicutes* were higher in patients with NAFL/NASH compared to healthy individuals.^488^ Development of hepatic steatosis in germ-free mice receiving transplants of gut microbiota from high-fat-fed mice demonstrates that the gut microbiome affects the progression of NAFLD.^489^ Patients with steatosis have a low microbial diversity, which is shown to associate with the decreased presence of “beneficial” *Coprococcus* and increased “harmful” *Ruminococcus Gnavus.*^477^ Gram-negative *Proteobacteria* are increased in advanced liver fibrosis, whereas Gram-positive *Firmicutes* significantly decreased, suggesting microbial profile might serve as a non-invasive marker for advanced fibrosis.^488,490^ It was reported that oral administration of *Faecalibacterium prausnitzii* indeed reduced liver fat content as well as ALT and AST levels, while FAO levels were elevated in mice fed with HFD.^491^ Oral administration of *Bifidobacterium longum* was able to significantly reduce blood glucose levels and TC levels and improve liver fat accumulation in mice fed with HFD.^492^ This positive effect may have been achieved by modulating the mRNA expression of components of the renin-angiotensin system.^492^ *Lactobacillus*, such as *L. acidophilus, L. fermentum*, and *L. plantarum*, reduced hepatic steatosis by lowering cholesterol in mice fed with a western diet.^493^ *Lactobacillus lactis* and *Pediococcus pentosaceus* also restored metabolite dysregulation in the gut of western diet‐induced NAFLD mice.^494^

Metabolites of the gut flora such as ethanol, short-chain fatty acids (SCFAs), and amino acids have also been demonstrated to be involved in regulating the disease process of NAFLD. In a Chinese cohort, high-ethanol-producing *Klebsiella pneumoniae* has been linked to up to 60% of patients with NAFLD.^495^ Excess alcohol produced by *Klebsiella pneumoniae* in the gut could enter the liver via the portal vein, causing hepatic lesions that was similar to the anatomy and histopathology of alcoholic fatty liver. Increased SCFAs were detected in the feces of patients with NAFL/NASH including acetate, propionate, and butyrate.^496^ Other studies, on the other hand, imply that SCFAs help to alleviate intestinal injury and reduce hepatic steatosis and inflammatory damage through various mechanisms. This apparent contradiction highlights the need of distinguishing between circulation and fecal SCFAs, as circulating SCFA levels are more closely linked to lipid metabolism and insulin sensitivity.^497^ Among them, butyrate has been most extensively studied and can improve NAFLD through various mechanisms. Sodium butyrate (SoB) prevented the progression of NAFL to NASH induced by HFD in mice by promoting hepatic GLP-1R expression.^498^ SoB can also alleviate hepatic steatosis in mice fed HFD through correcting the imbalance of gut microbiota and improving the intestinal barrier.^499,500^ Oral supplementation of SoB also showed efficacy in a diet rich in fat, fructose, and cholesterol or a western-style diet-induced murine NAFLD models.^501,502^ However, one study found that the gut microbiome of patients with NAFLD-associated liver cancer promoted butyrate production, and excessive butyrate could be detrimental to patients with liver cancer by blocking the normal immune system function.^503^ Amino acids that are fermented by intestinal microorganisms produce a number of metabolites, some of which exhibit protective effects on liver function. Glycine analogs (DT-109) with dual hypolipidemic/hypoglycemic properties attenuated experimental NAFLD by stimulating hepatic FAO and glutathione synthesis, inhibiting NF-κB target genes and TGFβ/SMAD signaling to diminish inflammatory infiltration and liver fibrosis, and may serve as potential therapies for NAFLD.^504^ The metabolism of tryptophan, an essential aromatic amino acid, produces indoles that enhance the barrier function of the intestine.^505^ Another tryptophan metabolite, indole-3-acetic acid, reduced the inflammatory response in the liver, downregulated adipogenesis-related genes, and alleviated steatosis in mice fed HFD. Other amino acids such as arginine and citrulline can also protect mice from the development of NAFL/NASH.^506–508^

Several amino acid metabolites, on the other hand, accelerate the development of NAFLD. Imidazole propionate is a microbially produced histidine-derived metabolite that is present in higher concentrations in patients with T2DM. Imidazole Propionate activates p38γ MAPK, which promotes p62 phosphorylation and subsequent activation of mTORC1.^509^ In patients with hepatic steatosis, the levels of N,N,N-trimethyl-5-aminopentanoic acid (TMAVA, a metabolite from intestinal bacterial metabolism of trimethyl lysine) in plasma were increased.^510^ TMAVA reduces carnitine synthesis and FAO and promotes HFD-induced steatosis in mice. Long-term treatment with phenylacetic acid, a metabolite of phenylalanine, successfully induced steatosis.^511^ In addition, ingested choline can only be converted to trimethylamine (TMA) by intestinal bacteria, and subsequently, TMA is converted to trimethylamine–N-oxide (TMAO) in the liver.^512,513^ The HFD enhanced choline catabolism of *Escherichia coli* and thus increased circulating TMAO levels.^514^ The level of circulating TMAO was positively correlated with the presence and severity of NAFLD,^515^ increased circulating TMAO inhibited hepatic FXR to exacerbate steatosis.^516^ These observations suggested that microbiota regulation targeting metabolic homeostasis and intestinal barrier is expected to provide new strategies for NASH treatment. The precise mechanisms of hepatic immunity mediated by flora metabolites and the elaborate regulatory network among metabolites need to be further investigated.

The intestinal barrier effectively prevents harmful intestinal bacteria and their metabolites from entering the circulation, which causes bacteremia or other tissue damage.^517^ Altered intestinal barrier triggers inflammatory response and remarkably affects the gut–liver axis. Increased intestinal permeability is found in patients with NAFL/NASH, accompanied by elevated ethanol-producing bacteria, which further promote the disruption of gut tight junction by stimulating the production of proinflammatory cytokines and correlate with the degree of hepatic steatosis.^518,519^ NAFL/NASH patients have elevated levels of endotoxin in their blood.^520^ High-fat feeding increases intestinal permeability^521^ and the proportion of LPS-containing microbiota in the gut.^522^ Alterations of gut microbiota and disruption of intestinal barrier increase the circulating levels of LPS.^523,524^ The levels of serum LPS are higher in patients with NAFL/NASH,^525^ and higher LPS hepatocyte localization is observed in patients with NASH.^526^ Subsequently, high levels of LPS recruited more TLR4-positive macrophages and activated TLR4 platelets.^526^ LPS activates TLR signaling in the liver, resulting in persistent and chronic inflammation and hepatocellular injury.^479,527^ Probiotic intervention reduced lowered plasma LPS levels and liver inflammation in turn delaying the progression of NAFL/NASH.^528^ Taken together, the gut–liver axis offers a new direction for the treatment of NAFL/NASH.

#### Gut microbiota targets

Gut microbiota manipulation yielded encouraging results for the treatment of different metabolic disorders in experimental models (Tables 3 and 4).^529^ Relevant clinical trials have been conducted (NCT02158351 and NCT04130321) with no results posted yet. Probiotics treatment may reduce intrahepatic triglyceride content from 22.6% to 14.9% (*p* = 0.034) and AST level (*p* = 0.008) in patients with histology-proven NASH (NCT00870012).^530^ Steatohepatitis was alleviated after fecal microbiota transplantation (FMT), as indicated by a significant decrease in intrahepatic lipid accumulation, intrahepatic proinflammatory cytokines, and the NAS score in mice.^531^ However, in a randomized, double-blind, placebo-controlled trial (NCT04074889), ultrasound-diagnosed NAFL/NASH patients supplemented with a probiotics sachet (MCP^®^ BCMC^®^ strains) containing six different *Lactobacillus* and *Bifidobacterium* species have no significant changes at the end of the study in terms of hepatic steatosis and fibrosis levels as measured by transient elastography.^532^ In further studies, more attention should be paid to the sample sizes, the duration of treatment, and different probiotic strains to evaluate the real benefits of probiotics in NAFL/NASH.

### Cell therapy

**Fig. 8:**
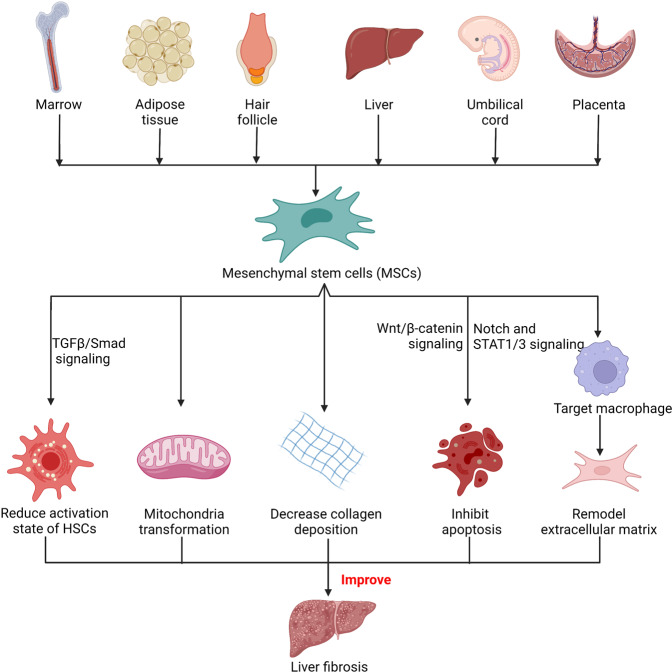
Stem cell therapy in liver fibrosis. MSCs isolated from different sources, including umbilical cord, bone marrow, placental, adipose tissue, placental, hair follicle, function to improve liver fibrosis. MSCs reduced hepatocyte damage through immune suppressive pathways, which in turn prevents HSC activation. Furthermore, MSCs increased the phagocytosis of hepatocyte debris by changing macrophage polarity, while increasing matrix metalloproteinase synthesis to remodel extracellular matrix. Created with BioRender

Cell treatment for NASH fibrosis has recently gained extensive attention. Mesenchymal stem cells (MSCs) have the potential for self-renewal and differentiation into multiple cell lineages, including hepatocytes. The migration and engraftation of MSCs into targeted lesions may exert clinical efficacy, such as the improvement of liver fibrosis in patients with NASH.^533^ It was reported that MSCs reduce hepatocyte damage through immune suppressive pathways, which in turn prevent HSC activation.^534,535^ Furthermore, MSCs increase the phagocytosis of hepatocyte debris by regulating macrophage polarity, while increasing matrix metalloproteinase synthesis to reduce ECM^534^ (Fig. 8).

It has been proposed that MSCs isolated from different tissues, including umbilical cord, bone marrow, placental, adipose tissue, placental, hair follicle, function to improve liver fibrosis. Infused human umbilical cord-derived mesenchymal stem cells (hUC-MSCs) are able to differentiate into hepatocytes in vivo, and transplantation of hUC-MSCs to CCl_4_-treated rats improved liver transaminase, reduced liver histopathology, and reversed hepatobiliary fibrosis.^536^ Exosomes of MSCs may also be a promising strategy for the treatment of NAFL/NASH. The hUC-MSC‑exosomal miR‑627‑5p improved glucose and lipid metabolism in a human normal liver cell line (LO2) and alleviated liver damage in high-fat high-fructose–fed induced NAFL rat model by repressing fat mass and obesity-associated gene expression.^537^ HLSC-Evs were shown to attenuate the activated phenotype of HSCs by delivering antifibrotic miRNAs, such as miR-146a-5p.^538^ Human adipose-derived MSCs or their EVs significantly increased anti-inflammatory macrophages in the liver and improved liver fibrosis.^539^

The therapeutic efficacy of MSCs mainly relies on the paracrine actions on different cell type, differentiation of MSCs, and inflammatory actions in the liver.^540^ In the preclinical studies, hUC-MSCs transplantation improves liver function, the degree of fibrosis, and promotes liver repair in acute-on-chronic liver failure/acute-on-chronic liver injury rats by inhibiting Notch signaling and reversing the imbalance of the Stat1/Stat3 pathway.^541^ Contrarily, adipose-derived stem cells (ADSCs) treatment reduces apoptosis of hepatocytes through Notch signaling activation and contributes to the repair and regeneration of the liver in NASH mouse model.^542^ The role of the Notch signaling pathway in the treatment of stem cell needs to be underlined.

In other novel studies with cell therapy implication, bone marrow-derived MSCs (BMSCs) were shown to regulate mitochondrial quality control, improve mitochondrial dysfunction, lower the AST/ALT ratio and alleviate the steatosis and histological lesions in livers of diabetic NAFL mice via mitochondrial transfer between stem cells and steatosis hepatocytes.^540^ BMSCs engrafted within the liver tissue prevented histological alterations and collagen accumulation via upregulating hepatic Nrf2/HO-1 signaling pathway in CCl_4_-intoxicated rats.^543^ In addition, BM-MSCs also attenuated hepatic fibrosis by secreting IL-4 and IL-10 to promote macrophage phenotypic switch from profibrotic Ly6C^hi^ subset to restorative Ly6C^lo^ subpopulation, which significantly blocked the source of fibrogenic cytokines (TGF-β, PDGF, TNF-α, and IL-1β) from Ly6C^hi^ macrophages.^544^ MiR-375 encapsulated by Evs secreted from BMSCs inhibited HCC development via modulating the HOXB3/Wnt/β-catenin signaling axis.^545^ ADSCs alleviated liver fibrosis in NASH mice by suppressing IL-17-mediated inflammation.^546,547^

In clinical trials, MSCs have also achieved promising results in the treatment of NASH. Umbilical cord-derived MSC (UC-MSC) treatment markedly improved liver functions, as indicated by the levels of serum albumin, prothrombin activity, cholinesterase, and total bilirubin after 48 weeks follow-up (NCT01220492). UC-MSC treatment is safe and does not increase the occurrence of HCC events, and it has a long-term effect on improving liver function and survival in patients with decompensated liver cirrhosis.^548^ In addition, CCR2 overexpression in MSCs improves the treatment effect of MSCs by enhancing the targeted migration of stem cells to damaged livers,^533^ and may provide a novel strategy for improving the efficacy of cell therapy for NASH.

### Immunotherapy for NASH-HCC

NASH is rapidly becoming one of the important drivers of HCC,^549^ and immunotherapy is considered to activate T cells or reinvigorate immune surveillance against cancer, with a response rate of 15–30% in patients with HCC.^550–553^ Recently, a study was performed to investigate the roles of immune checkpoint blockade (ICB) in NASH-HCC and non-NASH-HCC mice.^554^ It was observed that the liver tumors in NASH-HCC mice did not regress after anti-PD1 treatment, while tumors in non-NASH-HCC cancer models were shrunk by the same treatment. Unfortunately, liver fibrosis was exacerbated after anti-PD1 treatment in NASH-HCC models.^554^ Mechanistically, unconventionally activated CD8^+^PD1^+^ T cells accumulate progressively in NASH liver, but anti-PD1 treatment promotes the infiltration of CD8^+^PD1^+^ T cells population and secretion of TNF-α, leading to the increase of inflammation, fibrosis, and tumorigenesis.^554,555^ Cytotoxic CD8T^+^ cells that are suppressed in cancer accumulate in NASH and increase more after anti-PD1 therapy. The failure of PD1 therapy in NASH-HCC may be due to the loss of the original immune surveillance function of CD8T^+^ cells. Furthermore, a meta-analysis including three large randomized controlled phase 3 clinical trials of anti-PD(L)1 immunotherapy comprising over 1600 patients with advanced HCC (CheckMate-459,^556^ Imbrave150,^550^ and KEYNOTE-240^551^) revealed that although immunotherapy improved survival in the overall population, but not improve survival in patients with non-viral HCC.^554^ Notably, the authors investigated two other cohorts which recruited small groups of NASH-HCC patients and found that compared with other malignant tumor patients, the overall survival time of patients with NASH-HCC after immunotherapy was shortened. The abundance and complex signature of innate and adaptive immune cells in the liver may directly or indirectly affect the immunotherapy of NASH-HCC, which may cause less responsiveness to anti-PD1 immunotherapy.^554,555,557^ These observations suggest that ICB therapy may not be as effective as desired, future works might be required to better understand ICB resistance in NASH-HCC.

### Genetic approaches

Inherited factors have been proved to be associated with susceptibility to different stages of NAFL/NASH, suggesting that genetic mapping approaches might be a powerful tool to identify genes with diagnostic and therapeutic potential.^558^ In the DiscovEHR human genetics study, truncated loss-of-function variant in hydroxysteroid 17β-dehydrogenase 13 (*HSD17B13* rs72613567: TA), a lipid droplet-associated protein that is enriched in liver and hepatocyte with retinol dehydrogenase activity, was found to associate with decreased levels of ALT and AST and improved liver histology of NASH.^559^ The carriers of the *HSD17B13* variant have a lower risk of developing NASH and cirrhosis but increased phospholipids that is opposite to the characteristics of the choline-deficient model of liver fibrosis.^560^ Interestingly, *Hsd17b13* whole-body deficiency had no effect on protecting the liver from obesity diet damage in mice suggesting that interspecies differences need to be considered in the drug development process.^561^ However, some clinical trials in patients with NAFL/NASH have positive and hopeful results. ARO-HSD, a ribonucleic acid interference (RNAi) therapy, significantly reduced the mRNA and protein levels of hepatic *HSD17B13*, leading to a decrease in serum ALT in the phase 1 trial (NCT04202354).^558,562^

It was reported that *PNPLA3*-I148M variant was associated with hepatic steatosis, advanced fibrosis, and cirrhosis in patients with NAFL/NASH.^563^ The *Pnpla3*-I148M variant promoted steatosis by inhibiting adipose triglyceride lipase in mice fed high sucrose diet.^564^ The liver-targeted GalNAc3-conjugated antisense oligonucleotide (ASO)-mediated silencing of *Pnpla3* reduced hepatic lipogenesis and steatosis in mice carrying the human I148M mutant fed a NASH-inducing diet.^565^ Recently, ION839 (AZD2693), an ASO targeting PNPLA3, is treated to participants with NASH and homozygous for PNPLA3 I148M risk allele in a phase 1 clinical trial (NCT04483947). In addition, IONIS-DGAT2_Rx_, an ASO targeting diacylglycerol acyltransferase 2 (DGAT2, an enzyme that catalyzes the final step in TG synthesis in the liver), improved liver steatosis in patients with NASH in phase 2 clinical trial (NCT03334214).^566^ *GalNAc-siTAZ*, a stabilized TAZ (the transcriptional coactivator with PDZ-binding motif) siRNA bearing the hepatocyte-specific ligand N-acetylgalactosamine, partially reversed hepatic inflammation, injury, and fibrosis in mice.^567^ It is reported that the symptoms (fibrosis and hepatic glucose production) of NASH mice are improved by Notch inhibitors such as *Nicastrin* antisense oligonucleotide (*Ncst* ASO)^568^ and γ-secretase inhibitors (GSI).^455^ These results suggest that ASO or RNAi therapy as a genetic approach may have promising clinical applications for NAFL/NASH treatment.

### Combined therapies

Given the complexity of the pathophysiology of NASH, it will necessitate the involvement of multiple targets and pathways to improve outcomes of pharmacological intervention, which justifies combination therapies in the treatment of NASH. European Medicines Agency guidelines published in 2018 recommend that combination therapy should use drugs with different mechanisms of action.^569^ FXR agonists are most often combined with other agents in the treatment of NASH to minimize side effects and to increase the likelihood of success by targeting different metabolic pathways.^204^ The combination of Cilofexor (an FXR agonist) and Firsocostat (an ACC inhibitor) has shown more effective in reducing the NA) (*p* = 0.040) and minimizing dose-dependent complications of FXR activation (e.g., pruritus and LDL elevation) than Cilofexor used alone in patients with F3-F4 fibrosis or compensated cirrhosis in a phase 2b trial (NCT03449446).^570^

A randomized, placebo-controlled, multicenter phase 2b clinical trial will evaluate the safety and tolerability of Tropifexor (TXR, a non-BA agonist of FXR) and Cenicriviroc (a CCR2/5 antagonist, CVC) combination therapy in patients with NASH and liver fibrosis (NCT03517540). Dual antagonism of CCR2 and CCR5 by CVC may complement antisteatotic, anti-inflammatory, and antifibrotic actions of TXR. Given this, combination therapy is likely to show additional benefits compared with monotherapy.^571^ In addition, studies to evaluate Tropifexor alone or in combination with Licogliflozin (a dual SGLT1/2 inhibitor) in patients with NASH and liver fibrosis (NCT04065841) are recruiting. A preclinical study revealed that ZLY18 (a quadruple FFA1/PPAR-α/γ/δ agonist) significantly reduced steatosis, hepatocyte balloon, inflammation, and liver fibrosis in the NASH models, and represented a novel and highly promising quad FFA1/PPAR-α/γ/δ agonist warranting further investigation and development.^572^

## Conclusion and future perspectives

The increased prevalence of NAFL/NASH and global health burden have provoked concentrated research and interest in therapeutics for patients with NAFL/NASH. There are still no approved drugs for the treatment of NAFL/NASH, despite numerous pathophysiological mechanisms and genetic variants having been identified. However, several clinical trials have achieved promising improvement in histology-proven NAFL/NASH, which encourages researchers and pharmaceutical industries to further develop safe and durable therapeutic strategies. For example, antidiabetic agents showed some promising results in NAFL/NASH patients with T2DM; for those patients who had defined genetic variants, direct interventions can be made by targeted therapies such as ASO or RNAi. Furthermore, considering the complexity of the pathogenesis of NAFL/NASH, the synergistic use of drugs with different mechanisms and personalized treatment plan may better benefit patients, especially for the combination of lifestyle changes and pharmacological interventions. Novel drugs, such as ASO and RNAi, targeting gut microbiome and stem cell therapy, have also been demonstrated to have the potential for the treatment of NASH and need to be further explored. In addition, standardized clinical trials and tailored animal models should be thoroughly conducted to improve the clinical success of new therapeutic applications. In summary, future studies are needed to better understand the pathogenic mechanisms linking liver metabolism, response to inflammation, and injury in NAFL/NASH development, with the goal of safe and consistent improvements.
